# Complex Catalytic Materials Based on the Perovskite-Type Structure for Energy and Environmental Applications

**DOI:** 10.3390/ma13235555

**Published:** 2020-12-05

**Authors:** Florin Andrei, Rodica Zăvoianu, Ioan-Cezar Marcu

**Affiliations:** 1 Laboratory of Chemical Technology & Catalysis, Department of Organic Chemistry, Biochemistry & Catalysis, Faculty of Chemistry, University of Bucharest, 4-12, Blv. Regina Elisabeta, 030018 Bucharest, Romania; florin.andrei@inflpr.ro; 2 Interdisciplinary Innovation Center of Photonics and Plasma for Eco-Nano Technologies and Advanced Materials, National Institute for Laser, Plasma and Radiation Physics, 409 Atomistilor Street, 077125 Magurele, Romania; 3 Research Center for Catalysts and Catalytic Processes, Faculty of Chemistry, University of Bucharest, 4-12 Blv Regina Elisabeta, 030018 Bucharest, Romania

**Keywords:** perovskite, catalyst, water photodecomposition, methane combustion, pollutant photodegradation

## Abstract

This review paper focuses on perovskite-type materials as (photo)catalysts for energy and environmental applications. After a short introduction and the description of the structure of inorganic and hybrid organic-inorganic perovskites, the methods of preparation of inorganic perovskites both as powders via chemical routes and as thin films via laser-based techniques are tackled with, for the first, an analysis of the influence of the preparation method on the specific surface area of the material obtained. Then, the (photo)catalytic applications of the perovskites in energy production either in the form of hydrogen via water photodecomposition or by methane combustion, and in the removal of organic pollutants from waste waters, are reviewed.

## 1. Introduction

The 21st century has brought new challenges to scientists in solving the issues generated, on the one hand, by the increased demand for energy and, on the other hand, by the more stringent exigencies in environmental protection. In recent years we witnessed a continuous search to find more economically viable processes to generate cleaner energy starting from well known as well as from new renewable resources using more performant catalysts than conventional ones. In this respect, researchers have been focused on developing new catalysts and to perfect the existing ones in order to enhance the sustainability of energy-generating processes. An example illustrating the development of the research trends concerns the utilization of perovskite-type materials as catalysts for different processes involving energy production as well as removal of pollutants by oxidation processes. Complex oxides having an ABO_3_ perovskite-type structure are possible candidates for catalytic combustion, their potential as oxidation catalysts being studied for the first time approximately 50 years ago [1,2]. These oxide materials are very active, highly thermally stable and present low volatility. Due to these properties, they were considered as potential candidates to replace the expensive oxide-supported noble metals (especially platinum and palladium) which are currently the combustion catalysts used on a large scale. The utilization of perovskite-type materials for this aim allows surpassing the issues posed by the formation of noble metals volatile oxides or by their sintering at high temperatures [3,4,5].

Photocatalysis is considered the key of solving energy generation and environmental pollution problems since it can use sunlight, which is one of the cleanest energy sources. Perovskites are intensively studied as photocatalysts, for several reactions such as: generation of hydrogen by photodecomposition of water and total photo-oxidation of different organic pollutants [6,7].

The purpose of this literature review is to highlight the state of the art concerning the influence of the physico-chemical properties of perovskite-type materials on their catalytic and photocatalytic efficiencies, focusing mainly on photoelectrochemical water splitting, the photodecomposition of organic pollutants and the catalytic combustion of methane reactions. To this aim, the scientific information from more than 300 references published during the last 20 years has been screened.

## 2. General Aspects Concerning Perovskites Materials

### 2.1. Inorganic Perovskites

**Figure 1:**
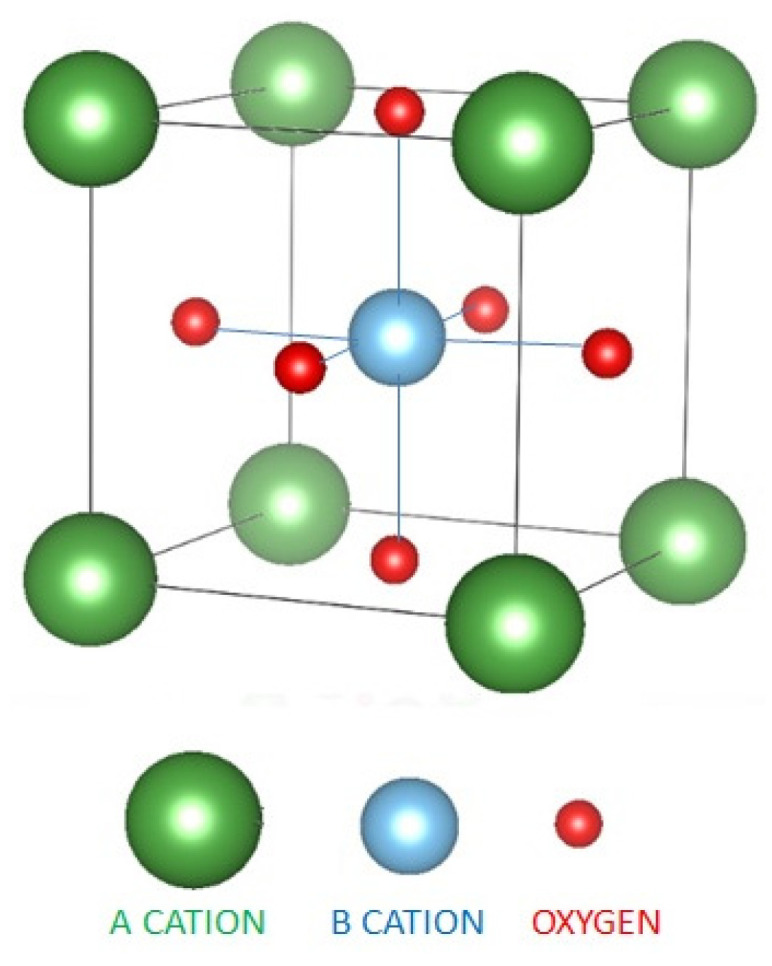
Inorganic ABO_3_ perovksite structure.

Most of the chemical compounds having the general formula ABO_3_ with r(A^n+^) ≈ 2r(B^m+^) (where r is the ionic radius of A and B cations with different valences forming compounds such as: A^I^B^V^O_3_, A^II^B^IV^O_3_ and A^III^B^III^O_3_) show an inorganic perovskite-type structure. Perovskites’ name derives from the first found mineral CaTiO_3_ which has the same atomic arrangement [8]. The unit cell of CaTiO_3_ is represented as a cube, where the corners are occupied by calcium ions, the titanium ions are positioned at the body’s center, and oxygen ions at the faces center (Figure 1). The name and the simple cubic structure were preserved even though in 1946 Megaw determined that CaTiO_3_ has an orthorhombic structure [9]. In the perovskite structure, the larger cation A is 12-fold coordinated with oxygen ions and responsible for the stability properties, having minor effects on the catalytic properties. Generally, the cations A are elements with inert d^0^ and f^0^ electronic structure, such as alkaline or rare earth cations. The catalytic activity is influenced by the smaller B cations with an octahedral coordination, which are usually 3d, 4d or 5d transition metals acting as active sites, because they have the ability to perform redox cycles without structure destruction [8,10].

In 1926, Goldschmidt [11] has defined a tolerance factor:

t = (r_A_ + r_O_)/2^1/2^(r_B_ + r_O_) (1)

where r_A_, r_B_ and r_O_ are the ionic radii of A and B cations and O anions, respectively, which is correlated to the thermodynamic and structural stability. The ideal perovskite cubic structure is stable only if 0.8 < t < 0.9, this range being slightly larger in the case of distorted perovskite structures. At room temperature, a distortion in the structure may exist, but at high temperatures, the cubic structure is formed. Besides the ideal cubic structure, other well-known symmetries for perovskites are: orthorhombic, rhombohedral, monoclinic and triclinic [12]. In catalysis, the perovskite stability during the catalytic cycles is an essential factor and it depends on the stability of the structural lattice, the valence stability of the transition metals in the reaction environment and, last but not least, the capability of defects’ formation [8]. Perovskites have the ability to incorporate mixed valance cations in their structure by either isostructural substitution of cations in a mother structure or by formation of structural anion vacancies, affecting in this way the structural and catalytic properties [8]. The most used substitutions involve the replacing of half of B cations with other cations having different charge (AB^x+^_0.5_B^y+^_0.5_O_3_). This type of replacement determines a shift of the oxygen ions present in the ordered structure toward the cations with higher valence. Moreover, the A and B cations in perovskites can both be easily substituted leading to doped compounds with the formula A_1-x_A’_x_B_1-y_B’_y_O_3_ [12]. Perovskite materials also show excellent properties for several applications, such as: dielectric, piezoelectric, semiconductors, electro-optic or superconductors [13]. Their utilisation as catalysts for energy production and environmental protection is detailed in Section 5 of this review.

### 2.2. Hybrid Organic-Inorganic Perovskites

**Figure 2:**
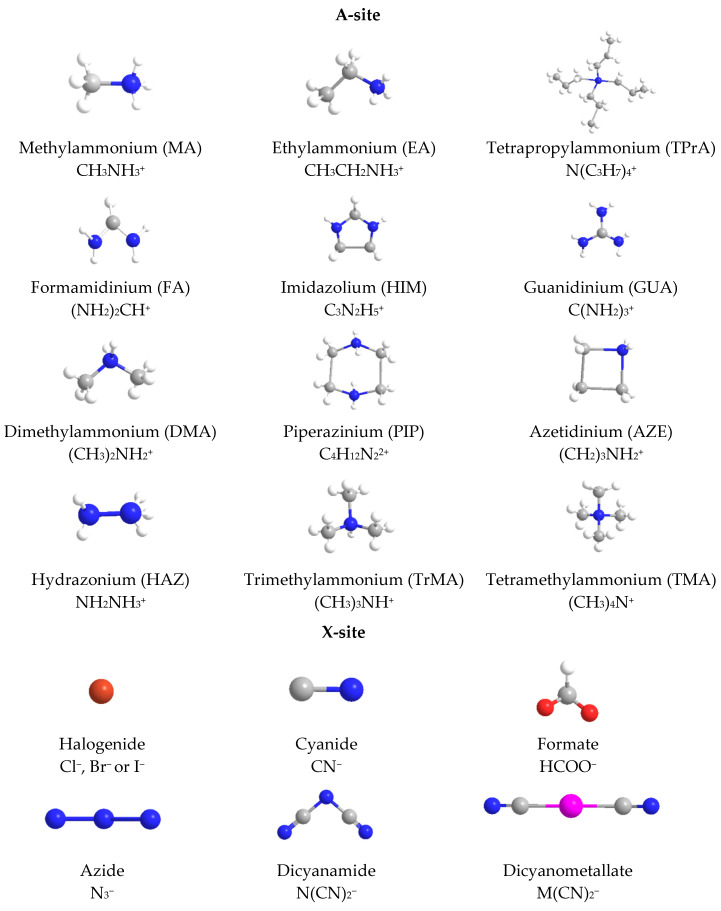
A-site and X-site ions of hybrid organic-inorganic perovskites. Adapted from reference [15].

Hybrid organic-inorganic perovskites, denoted HOIPs, are a class of materials derived from inorganic perovskites with general formula ABX_3_. Compared to the pure inorganic perovskite, in HOIPs the A site and/or X site are substituted by cations of organic amines and different inorganic/organic anions X, respectively. In this way, the rigidity and the compactness of the perovskite structure are diminished, and the organic part offers more functionalities and enhanced flexibility [14,15,16]. Several examples of A and X organic ions that can be integrated in a hybrid organic-inorganic perovskite structure are presented in Figure 2.

In 1978, the German researcher Dieter Weber reported for the first time the cubic phase of a hybrid perovskite (MA)PbX_3_, where MA is metylammonium and X is a halogen anion (Cl^−^, Br^−^ or I^−^) [16]. This structure shows an octahedral coordination around Pb^2+^ cations with halogen anions at the center of the faces of the cube, and the metylammonium cations occupy the A sites at the cube corners. Similar to the inorganic perovskites, the structure of HOIPs is defined by a Goldschmidt tolerance factor (t). The replacement of A and X sites in HOIPs with organic linkers leads to an adaption of this factor generating a formula with higher complexity, as follows:

t = (r_Aeff_ + r_Xeff_)/2^1/2^(r_B_ + h_Xeff_/2) (2)

where r_B_ is the ionic radius of B cation, r_Aeff_ is the effective radius of A cation and r_Xeff_, h_Xeff_ are the effective radius and the height of X anion modeled as a rigid cylinder, respectively. Many HOIPs show a tolerance factor ranging in the interval 0.8 < t < 1 [17].

The HOIPs are greatly utilized with excellent results for energy production as photovoltaics, while their efficiencies in catalytic applications are very low due to their instability. Considering the highlight of this scientific work and taking into account their instability in the reaction environment, this review will be focused only on inorganic perovskite materials.

## 3. Preparation of Inorganic Perovskite Materials

### 3.1. Preparation of Powders via Chemical Routes

#### 3.1.1. Co-Precipitation Method

One of the simplest and utilized synthesis methods for perovskite catalysts is co-precipitation. This method implies the precipitation of the metal precursors (oxides, alkoxides, nitrates or other inorganic salts), when the solubility limit decreases as an effect of adding a chemical reagent. Nitric acid can be used for dissolving the metal precursors having a low solubility [18,19], while an ammonium hydroxide solution can be used to adjust the pH in order to facilitate the precipitation [18,19,20]. The precipitate obtained is further aged, filtered and washed with deionized water until the salts in excess are completely eliminated. Finally, the resulting precipitate cake is dried, calcined and activated [21]. In order to obtain homogeneous products, it is necessary to precisely control some parameters, such as temperature, pH, coprecipitation rate and the concentrations of precursors. The main advantage of using this preparation method is that the resulting materials present higher specific surface area (SSA) than those prepared by other methods [22,23].

#### 3.1.2. Synthesis from Amorphous Precursors—“Citrate” Method

The “Citrate” method is a preparation technique which offers an extremely good control of the stoichiometry of reaction components. Moreover, it shows high reproducibility and an enhanced degree of homogeneity of the reaction mixture [24,25]. The principle of this method is based on a complexation reaction between metal cations (which were previously added in solution by dissolving specific metal nitrates in deionized water) and a chelating agent (generally citric acid, or ethylene glycol). The molar ratio between metal ions and complexing agent is 1:1 [26,27,28]. Other substances that can be used as chelating agents are: ethylenediaminetetraacetic acid (EDTA) [29], oxalic acid [30], tartaric acid [31] or glucose [32]. The resulting mixture is heated at 80–90 °C leading to a very viscous solution. The complex formed between metal cations and organic ligand is finally calcined to obtain the mixed oxide [26,27,31].

#### 3.1.3. Combustion Synthesis Method

In this method, stoichiometric amounts of metal nitrates of the desired cations are brought in aqueous medium together with urea (or glycine, citric acid, glucose), which is used as sacrificial fuel. Nitrate salts are used not only for the strong oxidizing character of NO_3_^−^ anions, but also for their high solubility in water. Urea is the most used organic fuel especially due to its low price [33,34]. Biamino and Badini have demonstrated on LaCrO_3_ that this process can be divided in two steps [35]. The first step is, in fact, the synthesis of the perovskite, which is an endothermic reaction, while the second step is the exothermic reaction between oxygen from nitrates and urea. Since the endothermic reaction, implying the transformation of nitrates into the desired oxides, requires a high amount of energy to be completed, the necessary energy is provided by the oxidation of urea. Finally, a stabilization treatment is applied and the catalysts are calcined for 4 h in air in order to remove all carbonaceous deposits [36]. This method can be used to form perovskite oxides having a nanometric particle size.

#### 3.1.4. Hydrothermal Synthesis

When applying this method, the metallic precursors are dissolved in water or are brought in the form of a slurry under high pressure and temperature conditions. An important advantage of this technique is that crystalline powders can be obtained without calcination. The particle size and shape can be modified by controlling the reaction temperature, pH and the reagent’s concentration [37,38,39].

#### 3.1.5. Solid State Reactions

By this method, the metallic precursors (generally nitrates, carbonates or oxides) can be mixed with enhanced accuracy of the stoichiometric ratios of cations. Practically, the reagents are ball milled in a milling container. The resulting material is dried at 100 °C and calcined at 600 °C for 4–8 h in air. Afterward, the product is ground and sieved in order to collect solid particles with similar sizes and further calcined for longer time (5–15 h) at higher temperature (1300–1600 °C). The final product is again ground and sieved to collect the granular fraction having the appropriate size for its further utilisation [40,41,42].

#### 3.1.6. Influence of the Preparation Method on the Specific Surface Area of the Perovskite-Type Materials

It is well known that the specific surface area (SSA) plays a crucial role in the catalytic processes. Depending on the preparation method of the catalysts, the porosity, size and shape of pores and the pore distribution are significantly different. An optimization of previously mentioned structural parameters can lead to the design of highly active catalysts [43]. Next, different classes of materials based on perovskite-type structure are presented taking into account their textural characteristics and their ability to be an efficient candidate for catalytic reactions.

The influence of the preparation method on the specific surface area of undoped and doped ABO_3_ perovskites with A = Ba, Y, Pb, La, Dy and B = Al, Cr, Ni, Cu, Ru, Ce, is resumed in Table 1. Barium and lead titanates prepared by a solid state reaction show a very small SSA (<1 m^2^/g) [44]. Similarly, pure LaCuO_3_ prepared by the same method show a very small SSA of about 0.6 m^2^/g [45]. A slight increase of the SSA is observed for neat LaCrO_3_ and LaNiO_3_, but it does not exceed 5 m^2^/g [45]. The high values of the calcination temperatures involved in the preparation of perovskites via solid state reaction are responsible for the small values of the SSA. The utilisation of high temperatures during the calcination procedure leads to non-porous materials with a non-uniformity of the particle shape and size, and with small SSA [46]. Undoped LaCrO_3_ and doped LaCrO_3_ with different contents of Mg were prepared by the citrate method. The generated SSA are similar in the range of 5–7 m^2^/g [47]. The increase of the SSA of pristine LaCrO_3_ prepared by citrate method compared to the one prepared by solid state reaction (see Table 1) can be correlated with the calcination temperature, which is much lower compared to the temperature used for the preparation via solid state reaction. The addition of MgO to the perovskite powder leads to an increase of the SSA up to 37 m^2^/g [48]. SSAs in the range of 4–21 m^2^/g were obtained by the citrate method for Fe and Co doped LaCuO_3_ perovskites [49,50]. Pure LaNiO_3_ obtained by the plasmochemical method, which is based on the injection of metallic precursors into a reactor with an air plasma having a temperature in the range of 4000–6000 K, shows an SSA of 17 m^2^/g [51]. The freeze-drying technique was used to prepare La_1-x_Sr_x_B_1-y_Ni_y_ with SSA in the range of 10–16 m^2^/g [52,53]. In this method, the solvent is directly sublimed from the solid ice into vapor, avoiding the formation of the liquid phase which can alter the morphological and chemical homogeneity of the final product [54].

In Table 2 the influence of the preparation method on the specific surface area of lanthanide cobaltate-based perovskites is resumed. Generally, the co-precipitation method is used for both pure and doped cobaltates having rare-earth elements in the A site. The specific surface area of perovskites prepared by this method ranges from 1.6 to 8 m^2^/g [3,56,57]. The calcination temperature of the cake (precipitate), the calcination time and the heating rate are experimental factors controlling the specific surface area. Similar values of SSA were obtained by using the same preparation method for PrCoO_3_, NdCoO_3_ and GdCoO_3_, respectively [56]. The citrate method is a suitable technique for cobaltate perovskites with higher specific surface area. It is frequently used for both pure and doped complex perovskites. The specific surface areas are extended over a larger range starting from 6 m^2^/g for pure LaCoO_3_ and reaching a value of 18 m^2^/g for Ce-doped LaCoO_3_ [50,58,59]. Solid state reaction offers a smaller range of specific surface areas with a maximum value of 5.1 m^2^/g for Ba-doped LaCoO_3_ (20% of A site). Moreover, a slight increase of SSA with the increase of the ionic radii of dopants (Ca^2+^, Sr^2+^, Ba^2+^) is observed [45]. Several microstructured powders of different perovskite-type materials based on the La_1-x_Sr_x_Co_1-y_B’_y_O_3_ formula prepared by freeze-drying method were reported to show SSA ranging from 10.4 to 22.7 m^2^/g. Yamazoe et al. reported that the freeze-drying method allows the formation of higher surface area materials compared to other thermal evaporation techniques due to its great control of evaporation processes and lower decomposition temperatures [60]. A much higher value of SSA was reported for La_0.9_Ce_0.1_CoO_3_ prepared by flame pyrolysis (62 m^2^/g) compared to the same perovskite prepared by the citrate method (10 m^2^/g) [58,61]. This method implies the combustion in the flame of a solution containing the metallic precursors. It is a versatile technique which ensures an excellent control of the material crystallinity and particle size through its experimental parameters [62].

Manganates represent a class of perovskite materials having manganese in B site positions (AMnO_3_). The effect of the synthesis method on the SSA of lanthanide manganate-based perovskites is presented in Table 3. Compared to cobaltates, the SSA of manganates prepared by co-precipitation are higher with a maximum value of ca. 15 m^2^/g for pure LaMnO_3_ [3]. For manganese-based perovskites, one of the highest values of the SSA (68 m^2^/g) was obtained by the citrate method, which consisted of adding citric acid and ethylene glycol to a solution containing lanthanum and manganese nitrates. It has been observed that the specific surface area increases with the citric acid etching time [66]. A detailed study concerning both the neat and Mn-doped LaAlO_3_ perovskites reported that SSA increases with the Mn content reaching a maximum value of 33 m^2^/g for LaAl_0.2_Mn_0.8_O_3_. When aluminum cations are completely replaced with manganese cations, SSA decreases to 22 m^2^/g [55]. Pure LaMnO_3_ prepared by flame-pyrolysis shows an SSA of 56 m^2^/g. By substituting (10 at.%) of La cations with Sr^2+^, a slight decrease of the SSA can be observed (51 m^2^/g). By increasing the dopant concentration (20 at.%), the SSA increases up to 70 m^2^/g. In contrast to Ce-doped cobaltates prepared by flame-pyrolysis (62 m^2^/g), La_0.9_Ce_0.1_MnO_3_ shows a higher SSA of about 84 m^2^/g, with a particle size of 30–45 nm [61]. Yu et al. reported an accurate study concerning the effect of the preparation method on the SSA of Pd-doped LaMnO_3_ [67] showing that an inappropriate ratio between the metallic precursors and the organic fuel in the combustion synthesis leads to a very low SSA (1 m^2^/g), while higher SSA can be obtained by using amorphous citrate (12 m^2^/g) and flame pyrolysis (32 m^2^/g) methods. The ultrasonic spray combustion method generates Pd-doped LaMnO_3_ perovskite material showing the highest SSA (39 m^2^/g). In contrast to the classical combustion synthesis, this method uses an ultrasonic spray gun to initiate the reaction [68]. Notably, the crystal structure of the perovskite depends on the preparation method as well. Indeed, a rhombohedral crystal structure was obtained via ultrasonic spray combustion and citrate methods, while combustion and flame pyrolysis methods generated an orthorombic perovskite structure [67]. Ciambelli et al. reported the preparation of Ce- and Y-doped rare earth manganates by the mechanochemical method. Starting from oxide and carbonate precursors, the resulting materials showed SSAs of 14 and 19 m^2^/g, respectively [51].

Table 4 shows the effect of the synthesis method on the SSA for lanthanide ferrite-based perovskites. The co-precipitation method leads to LaFeO_3_ (21 m^2^/g) solids having even higher SSA than manganates (15 m^2^/g) and cobaltates (8 m^2^/g) [3,57,76]. The citrate method is one of the most used preparation techniques for pure LaFeO_3_ and doped LaFeO_3_ perovskites. Pure LaFeO_3_ showing the SSA of 2.9 m^2^/g was prepared by this method. It was observed that the SSA increases with the concentration of Mg^2+^ used as B-site dopant. This tendency is maintained for a maximum Mg^2+^ concentration of 40 at.%. A further increase of the dopant content leads to the reduction of SSA [77]. A similar value of the SSA for neat LaFeO_3_ prepared via citrate method was reported elsewhere [78]. No changes of the SSA were observed for La_0.7_Ca_0.3_FeO_3_ compared to simple LaFeO_3_. However, when increasing the Ca^2+^ content to 50 at.%, the SSA dramatically decreases down to 0.7 m^2^/g. Other publications report values of SSA of ca. 20 m^2^/g for undoped LaFeO_3_ prepared by the citrate method. Also, it increases to ca. 38 m^2^/g for Ca-doped LaFeO_3_ [76,79]. The substantial differences between the SSA of the same perovskite materials can be correlated to the calcination temperature used during the preparation procedure. Materials with smaller SSA were calcined at 800 °C for 5 h, while the others were obtained at 700 °C in 6 h [76,79]. Pd-doped LaFeO_3_ shows similar values of SSA as Pd-doped LaMnO_3_, when prepared via citrate and combustion methods. However, the SSA decreases for Pd-doped LaFeO_3_ prepared by flame pyrolysis and ultrasonic spray combustion methods. In contrast to Pd-doped LaMnO_3_, the formation of the orthorombic crystal structure of Pd LaFeO_3_ is independent on the preparation method [67]. Also, the mechanochemical method is used for the preparation of doped ferrites. The resulting materials possess SSA smaller than 10 m^2^/g [51].

The effect of the preparation method on the SSA of supported perovkites is resumed in Table 5. The wet impregnation technique was used to stabilize commercial γ-Al_2_O_3_ (having initial SSA of 200 m^2^/g) with 5 wt.% La_2_O_3_. Lanthanum nitrate, manganese acetate and urea were used to load the previously prepared support with 30 % LaMnO_3_ by using the deposition technique. This method is based on the precipitation of the active phase, which in this case is LaMnO_3_ perovskite, to the support surface. Finally, the sample was dried at 120 °C and then calcined in air for 3 h at 800 °C. The resulting material shows an SSA of 88 m^2^/g. Yet, the calcination temperature is not enough to obtain a pure perovskite, tiny amounts of La_2_O_3_ and La(OH)_3_ side-phases being observed by X-ray diffraction. MgO-supported LaMnO_3_ with an SSA of 25 m^2^/g was also prepared by the same method [70]. La_0.8_Sr_0.2_MnO_3_ was successfully dispersed on different MAl_2_O_4_ spinels supports (M = Mg, Ni, Co) via wet impregnation technique. The perovskite loading was 20 wt.% and the SSA varies between 18 and 34 m^2^/g [73]. Similar SSA were obtained for Ag-doped LaMnO_3_ loading metallic foils made of Fe-Cr-Al using the same impregnation technique. Before the impregnation, the metallic foil was washcoated with 84.8% Al_2_O_3_, 14.4% TiO_2_, 0.8% La_2_O_3_ through the citrate method [80]. Neat LaFeO_3_ prepared via citrate method was dispersed on similar supports, leading to materials with SSA of 7.7 m^2^/g [81]. The highest specific surface area was obtained for ZrO_2_-supported LaMnO_3_ (132.5 m^2^/g) [82].

### 3.2. Thin Films Manufacturing Using Laser-Based Techniques

#### 3.2.1. Pulsed Laser Deposition (PLD)

**Figure 3:**
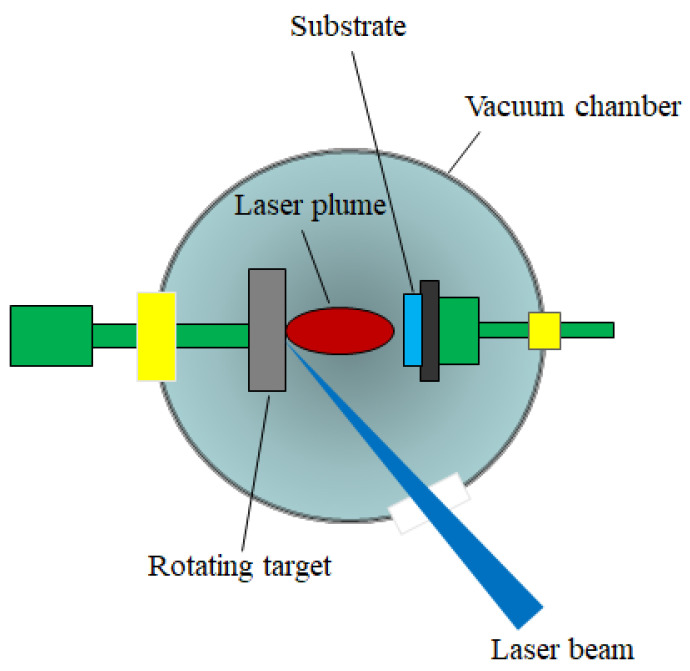
Pulsed laser deposition (PLD) technique scheme Adapted from Ref. [84].

Pulsed laser deposition (PLD) is a technique belonging to the physical vapor deposition (PVD) class which takes place in a vacuum chamber. The PLD setup is schematically presented in Figure 3.

**Figure 4:**
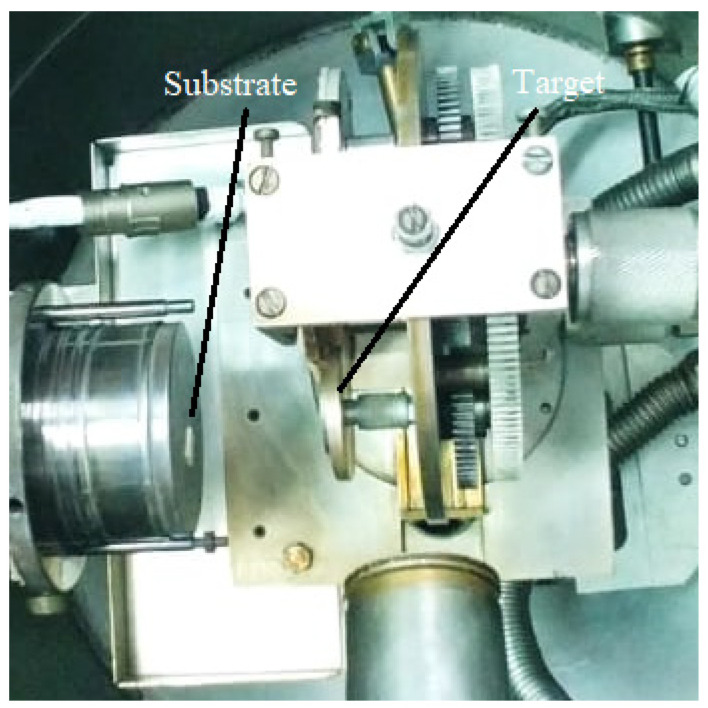
Photograph of the PLD setup: the heated substrate and the material target.

A pulsed laser beam is focused onto the surface of a target consisting of the desired material to be deposited. When the laser pulses have high enough energy density, a plasma plume forms at the target surface as an effect of vaporizing or ablating small parts from it. The flux of material necessary for the film growth is provided by the ablation plume and it is collected on a substrate. In other words, the deposition process using laser ablation is based on the vaporization of the target material, followed by the deposition of the vaporized material onto a collecting substrate. The substrate is situated at a well-known distance from the target and it is placed parallel to the target, as can be seen in Figure 4 [85,86].

**Figure 5:**
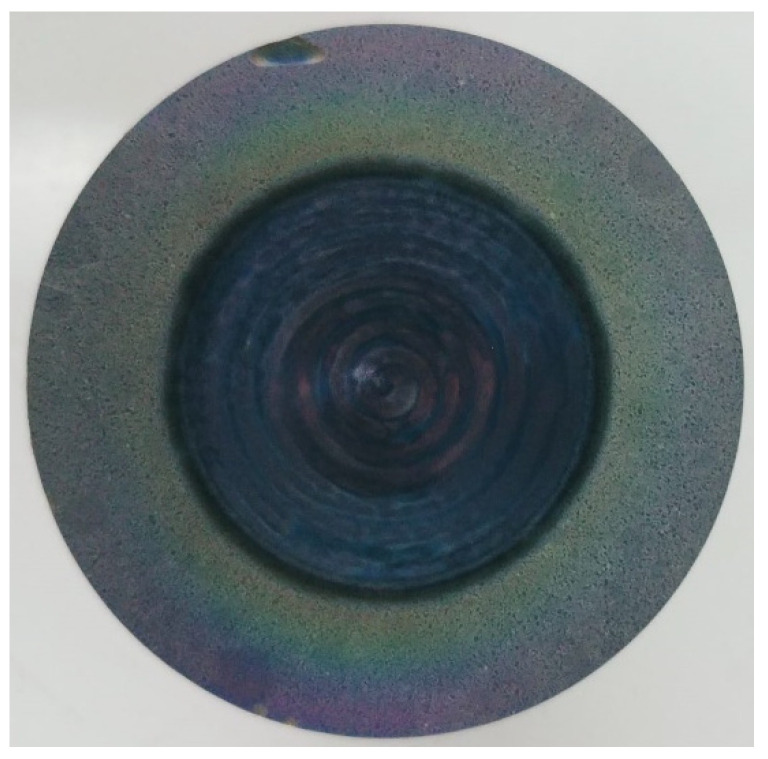
Photograph of a TiO_2_ target.

The fundamental processes taking place during the laser ablation are as follows: the heating of the irradiated material; the melting, evaporation or sublimation of the heated material; the formation of the plume and, finally, the plume expansion. One of the most important ablation parameters is the energy density of the laser pulse or its fluence (J/cm^2^). The preparation of thin films by using PLD can be performed both in a vacuum and in a gas atmosphere, which influences the deposition process. Figure 5 displays a photograph of a TiO_2_ target [85,86].

Pulsed laser deposition is a non-conventional technique, relatively simple and suitable for the production of thin films that can be successfully applied for many classes of materials when other techniques fail. It presents the following advantages:

- the laser radiation can be well focused on very small spot sizes at the target surface, increasing in this way the efficiency, the control and the flexibility of the process;
- the deposition chamber can be considered a “clean reactor” because the energy source (laser) is external, being independent of the deposition medium; also, the laser parameters (energy density and wavelength) can be easily adjusted to ensure the reproducibility of the sample preparation;
- it is a simple and versatile technique from the point of view of experimental achievement, offering the possibility to obtain all kind of materials (complex stoichiometry, organo-metallic compounds);
- the properties of the obtained thin films (thickness, crystalline structure, stoichiometry and composition) can be rigorously controlled, because they depend on the laser parameters (wavelength, laser fluence, the spot area, the duration of pulse, the repetition rate etc.) which are easily controlled from the outside of the deposition chamber;
- it ensures large deposition rates (1–5 Å/pulse).

However, the pulsed laser deposition technique has also a number of disadvantages, such as:

- the possibility to cover only substrates having small area (~1 cm^2^);
- the appearance of material droplets or clusters on the surface of the thin films leading to an increased roughness, which can affect the crystallinity, the optical, electrical and magnetic properties of the manufactured thin films [85,86].

The latter disadvantage can be diminished or even eliminated by optimizing the PLD system and deposition conditions as follows:

- the selection of a suitable target material: a target made by dense and very small particles, ensures uniform conditions during the ablation process. A material presenting a lot of defects or different structural mechanical strains, which can appear during the processing procedure, affects the deposition process. Moreover, the target material has to present a high absorption coefficient at the used laser wavelength;
- the rotating and the translation of the target material toward the laser beam during the deposition process;
- the optimizing of the deposition parameters (the laser fluence, the laser spot area, the repetition rate);
- the utilization of a supplementary laser beam parallel to the substrate surface which can split the material clusters.

**Figure 6:**
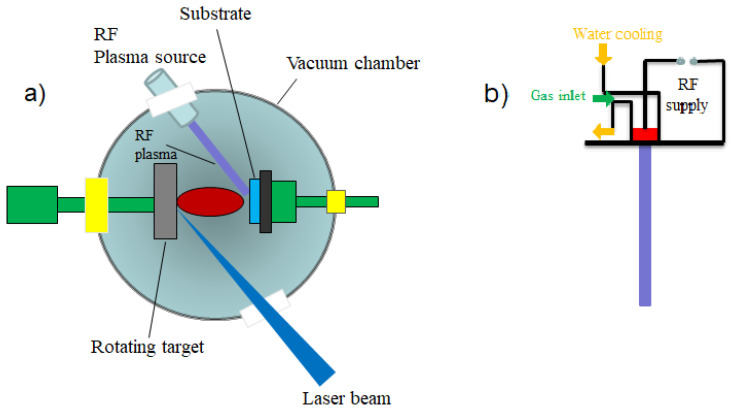
The experimental setup of radiofrequency (RF)-assisted PLD (**a**) and RF plasma source (**b**) Adapted from reference [86].

Additionally, the PLD can be coupled with a radiofrequency (RF) plasma source for better performance. Compared to the standard PLD setup, in this case a RF plasma source directed to the substrate is added. The function of the RF source is to ensure a supplementary control of the anionic composition of the manufactured thin films by using gases from the RF plasma source [87]. Figure 6 shows the experimental setup of the RF-assisted PLD vacuum chamber.

In Table 6 the experimental conditions for different types of perovskite materials grown by PLD and PLD-RF techniques are presented.

#### 3.2.2. Matrix-Assisted Pulsed Laser Evaporation (MAPLE)

**Figure 7:**
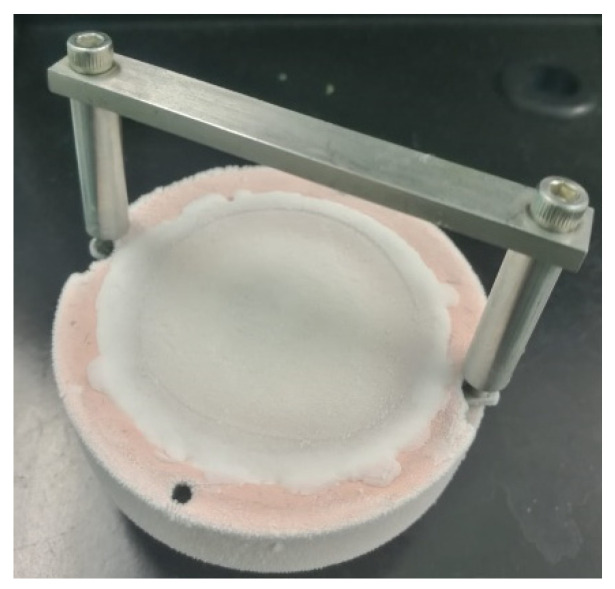
Photographs of frozen material used as matrix-assisted pulsed laser evaporation (MAPLE) target.

A major disadvantage of the manufacturing of thin films by laser ablation arises from the deposition process. The plasma formation and the condensation of elements on a substrate are not suitable for soft organic and polymeric materials, because their structure can be decomposed easily or even completely destroyed as an effect of interaction with laser beam. In order to depose this kind of materials, a modification of the experimental setup is required, this modification being related to the deposition target. Therefore, in the matrix-assisted pulsed laser evaporation (MAPLE) technique the target is prepared by dissolving the polymeric (or organic) material into a volatile solvent and the resulting mixture is frozen in liquid nitrogen. A photograph of a MAPLE target can be seen in Figure 7. The laser wavelength is selected in such a way that only the solvent reacts when the laser beam hits the frozen target [143,144].

**Figure 8:**
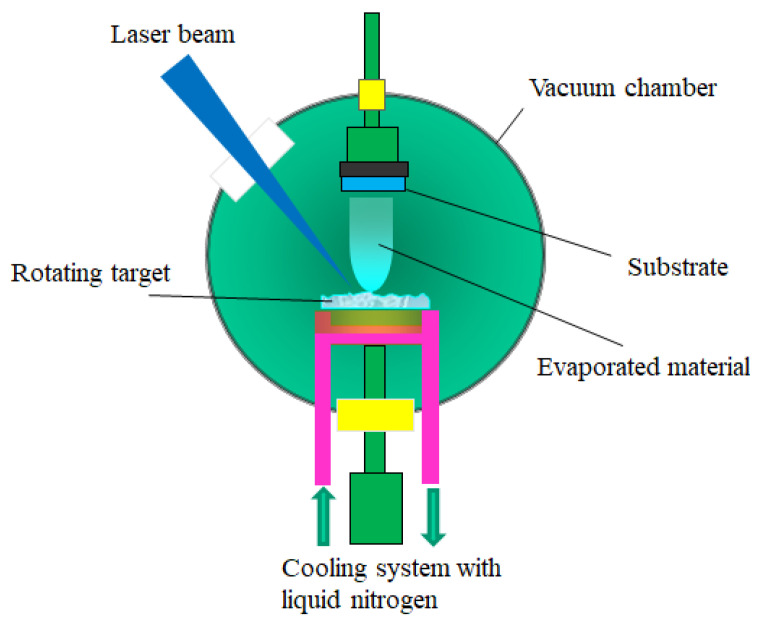
The schematic setup of the MAPLE technique. Adapted from reference [86].

The difference between this technique and PLD lays in the target preparation, which generates other laser-material interaction mechanisms [145]. The setup used for MAPLE deposition is presented in Figure 8.

In this case, two main processes occur when the laser beam falls onto the target surface: the evaporation of the frozen target and the ejection of the organic material. Generally, the concentration of polymeric (or organic) substance is very low (ca. 1–5%), the evaporation of the solvent and of the desired substance taking place simultaneously. The energy of the photons absorbed by the solvents is converted into thermal energy which induces the heating and the evaporation of the target. The polymeric chains have sufficient kinetic energy to cross the distance between the target and the substrate, while the solvent molecules are eliminated from the reaction chamber by the vacuum pumps. When the experimental conditions are optimized, the polymeric (or organic) substances can be transferred from the target to the substrate without structural damage [146,147]. This method has also been used to obtain some perovskite thin films (Table 7).

## 4. Catalytic Applications of Perovskite-Type Materials

### 4.1. Energy Production

Nowadays, one of the major problems facing humanity is to fully cover the global energy demands which have severely increased in the last few years. Most energy is generated by using fossil fuels. The high energy demand on the market accelerates the consumption of these exhaustible resources based on carbon. Moreover, the intense utilization of fossil fuels causes the greenhouse effect and generates huge amounts of pollutants affecting the environmental safety and human’s health [151]. Hence, a new alternative for energy production must be urgently and efficiently implemented. One of the most promising ways to solve the current energy problem is to use solar energy. The energy provided by the sun is abundant, among the cleanest energy resources, which does not make the global warming status worse. Furthermore, solar energy is an ecological and renewable source, and these features are propelling it as a suitable candidate for the global supply. Solar energy can be converted to both electrical and chemical energies by using photovoltaic and photocatalytic concepts. However, until technologies based on these processes are suitable for industrial implementation, new alternatives in order to increase fossil fuels’ efficiency and to minimize the pollution are required. Among others, catalytic combustion of methane is one of the promising ways to increase the efficiency and to minimize pollution [152].

#### 4.1.1. The Production of Energy in the Form of Hydrogen via Water Photodecomposition

Hydrogen is considered to be one of the most suitable options to replace the carbon-based fuels. It can be generated using different methods from various renewables (hydro, solar) and non-renewables (coal, natural gas, nuclear) sources. Among these, hydrogen can be produced from water through different processes, such as high-temperature decomposition (thermochemical water splitting) [153,154,155], electrolysis [156], photocatalysis [157] and photoelectrochemical water splitting [158]. Today, hydrogen is mainly synthesized by steam reforming of hydrocarbons (especially methane). It finds uses generally in petroleum refining [159], the production of ammonia [160] and in the metal refining industry [161]. For the future, it is intended to use hydrogen in fuel cells for high-efficiency power production systems which can directly produce electricity at low temperatures, with no emission of toxic by-products. In practice, inside a fuel cell, the hydrogen or a hydrogen-rich fuel is reacting with pure oxygen or oxygen from air, the resulting product being water [162]. As already discussed in Section 4.1, the solar energy is considered the most suitable alternative as global energy supplier and thus the water photodecomposition reaction is one of the most studied methods for chemical energy (hydrogen) production.

**Figure 9:**
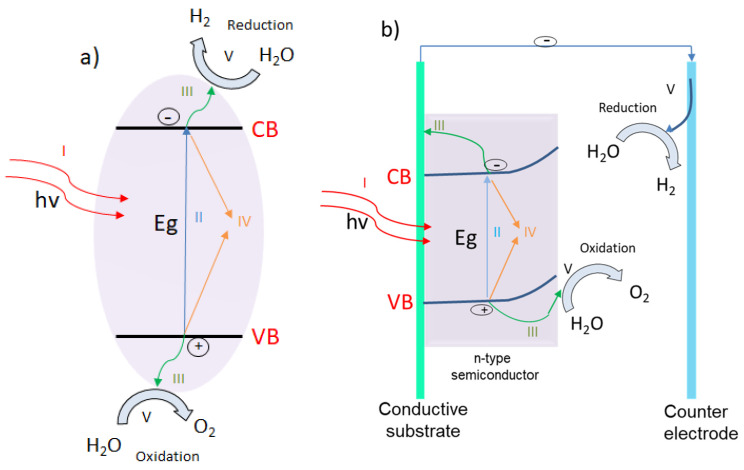
Schematic illustration of (**a**) photocatalytic (PC-I. Light absorption; II. Electrons jump from the valence band (VB) to the conduction band (CB); III. Redox processes; IV. Bulk recombinations) and (**b**) photoelectrochemical (PEC-I. Light absorption; II. Electrons jump from VB to CB; III. Transfer of charge carriers to the electrodes surface; IV. Bulk recombinations; V. Redox processes) processes Adapted from Ref [163].

There are two similar processes for the photodecomposition of water: photocatalysis, which is based on a particulate system, and photoelectrocatalysis (also known as photoelectrochemistry) based on photoelectrochemical (PEC) cells. A schematic representation of the mechanisms involved in these two processes can be found in Figure 9.

**Figure 10:**
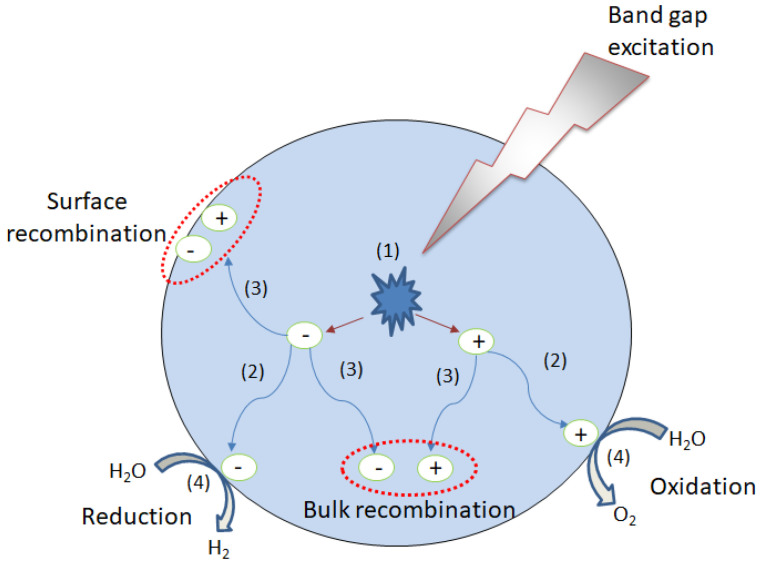
Schematic diagram of the charge carriers formation in a photocatalytic process. Adapted from reference [165].

Generally, in photocatalysis, the photocatalyst powders are freely suspended in a solution (as a slurry) or are fixed in a reactor bed. This is considered one of the most simple and cheapest methods for water photodecompositon which does not require transparent electrodes or directional illumination. As can be observed from Figure 9, the semiconductor photocatalyst particles are irradiated and the photons having energies higher than the material band gap are absorbed. The photons’ absorption is followed by the charge carriers formation, electrons (e^−^) are excited to the conduction band (CB), positive holes (h^+^) being simultaneously created in the valence band (VB). Once formed, the charge carriers migrate to the photocatalyst surface. When they arrive to the solid photocatalyst/liquid electrolyte interface, electrons are reducing water to molecular H_2_, while positive holes are oxidizing it to O_2_. Unfortunately, only a small portion of the photogenerated charge carriers participate in the redox reaction, and most of them suffer bulk or surface recombination, as can be seen in Figure 10. In fact, the charge recombination is one of the biggest challenges of this type of process, because a high loss of the excited charge carriers leads to a decrease in the reaction yield [164,165].

In order to evaluate the performance of a photocatalyst, the concept of the apparent quantum yield (QE %) was introduced and it is described by the following formulae [166,167]:

QE (%) = (2 × number of H_2_ molecules/number of incident photons) × 100 (for hydrogen) (3)

QE (%) = (4 × number of O_2_ molecules/number of incident photons) × 100 (for oxygen) (4)

In addition to the high recombination of the photogenerated charge carriers, the photocatalytic system has other major disadvantages. The photogenerated hydrogen and oxygen gas are formed on the same photocatalyst surface and, hence, they are predisposed to form water via the so-called “surface back-reaction” (SBR). In order to avoid this reaction, the photocatalytic systems require the utilization of additional sacrificial reagents which are electron acceptors or donors. Moreover, the final separation of the gas produced requires additional energy consumption. Also, because the solid particles are suspended in the liquid electrolyte, a part of irradiation light will be absorbed by the liquid medium, decreasing the energy of incident light which interacts with the photocatalyst [167].

**Figure 11:**
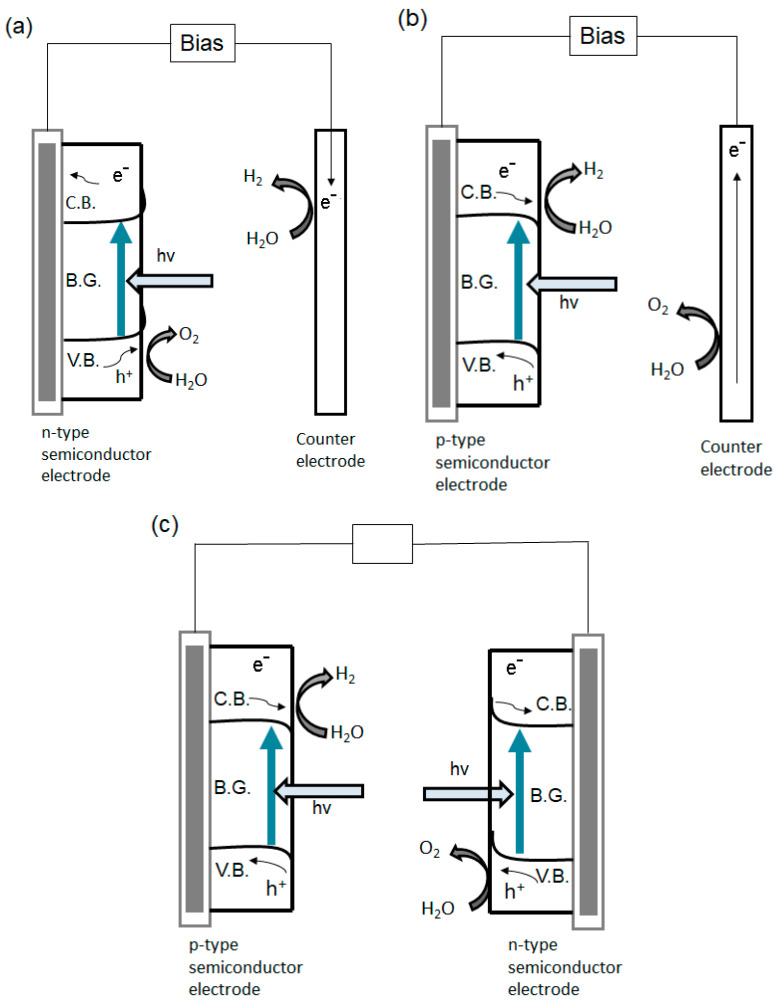
Photoelectrochemical water-splitting systems with: (**a**) n-type semiconductor photoanode; (**b**) p-type semiconductor photocathode; (**c**) tandem system. Adapted from reference [163].

On the other hand, in a PEC system the standard configuration is based on a photoelectrode (called working electrode) and a counter electrode, as presented on Figure 9. The working electrode consists of a semiconductor material (of either n-type or p-type) deposited on a conductive substrate. When the semiconductor material is of n-type, the working electrode acts as photoanode (Figure 11a), while when it is of p-type, it operates as photocathode (Figure 11b). Moreover, the PEC system can also work as a tandem system with both photoanode and photocathode, as shown in Figure 11c.

For example, in a standard PEC configuration, when photons are absorbed by a n-type semiconductor, the positive holes from the valance band are migrating to the photoelectrode surface, where water is oxidized to O_2_. The electrons are collected by the conductive substrate and they are sent through an external circuit to the counter electrode (Pt), where water molecules are reduced to H_2_. Generally, in order to ensure a better separation of the charge carriers, an external electrical or chemical (pH difference) bias between electrodes is applied. When the working electrode is made of p-type semiconductor, O_2_ is formed at the counter electrode surface, while H_2_ is generated on the p-type photoanode surface. Also, the same principle is applied for tandem PEC configuration, water molecules are reduced to H_2_ at the p-type semiconductor surface while being oxidized to O_2_ at the surface of the n-type semiconductor [163].

The PEC system shows excellent advantages compared to the particulate system, the oxygen is generated at the photoanode, while the hydrogen is formed at the photocathode. In this way, the resulting gas can be independently collected with no further costly gas separation methods or efficiency lowering because of SBR. Furthermore, appropriate and superior quality contact between the conductive substrate and semiconductor ensures excellent charge transfer leading to great photoelectrochemical efficiencies [168].

**Figure 12:**
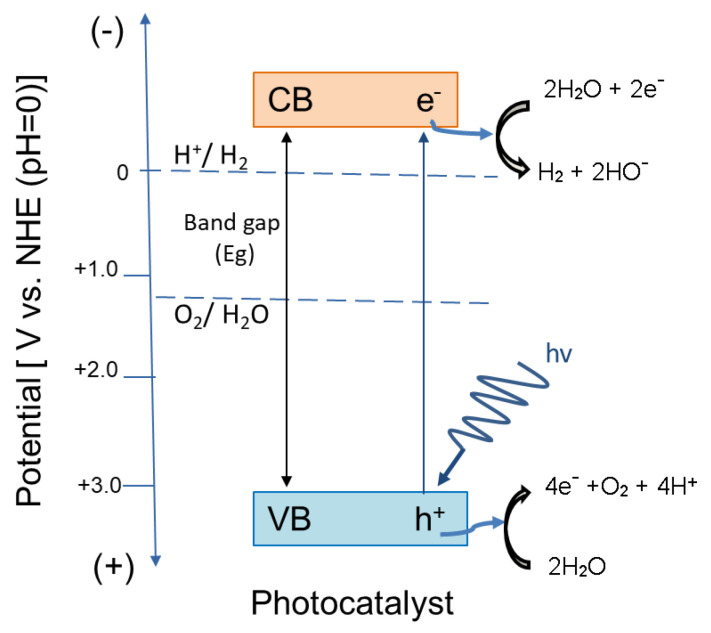
The ideal VB and CB positions for water splitting reaction. Adapted from reference [168].

The PEC water-splitting processes can be divided into two electrochemical redox reactions: the hydrogen evolution reaction (HER) and the oxygen evolution reaction (OER). The HER can occur by the Volmer–Heyrowsky or Volmer–Tafel mechanisms following a two-electron transfer process (2H_2_O + 2e^−^ ⇄ H_2_ + 2HO^−^) [169]. On the other hand, the OER is a more complicated redox process implying the transfer of four electrons (2H_2_O ⇄ 4e^−^ + O_2_ + 4H^+^) [170,171]. Depending on the experimental conditions, as well as, the nature and the physico-chemical properties of the photoelectrodes, the backward reactions can affect the global water splitting efficiency. The hydrogen oxidation reaction (HOR) is based on the oxidation at the cathode of H_2_ to H^+^ by the same mechanism as HER. By contrast, at the anode, the oxygen reduction reaction (ORR) can directly or indirectly occur by the same four-electron transfer process. The direct reduction of O_2_ leads to H_2_O formation, and it can be associative or dissociative. On the other hand, the indirect process leads to water formation from intermediate compounds such as hydrogen peroxide [172,173]. The dissociation of H_2_O molecule into H_2_ and ½O_2_ is a thermodynamically non-spontaneous reaction and, hence, in order to occur, it requires an excess of energy corresponding to a Gibbs free energy of +237 kJ/mol H_2_ (1.23 eV/electron transfer). In other words, the water photo-dissociation can theoretically occur only if the absorbed photons have energies higher than 1.23 eV, which correspond to 1100 nm. As can be seen, the infrared light is the most suitable for water photodecompositon reaction, at least from a theoretical point of view. Nevertheless, in practice a higher energy is required because of the energy losses (electron/hole transfer, the recombination of electron/hole, kinetic losses). Generally, it is recommended to use photocatalysts/photoelectrodes having a band gap value of 2.0–2.2 eV to overcome losses. Another important factor for the overall efficiency of water splitting reaction is related to the energy position of the bands extrema. In order to make possible the HER the bottom of the conduction band (CB) of the photocatalyst must be located at a potential which is more negative than the reduction potential of H^+^/H_2_ (0 V vs. NHE—normal hydrogen electrode) and the top of the valence band (VB) has to be located at more positive potential than the oxidation potential of H_2_O/O_2_ (1.23 V vs. NHE) [174]. A schematic representation is presented in Figure 12.

**Figure 13:**
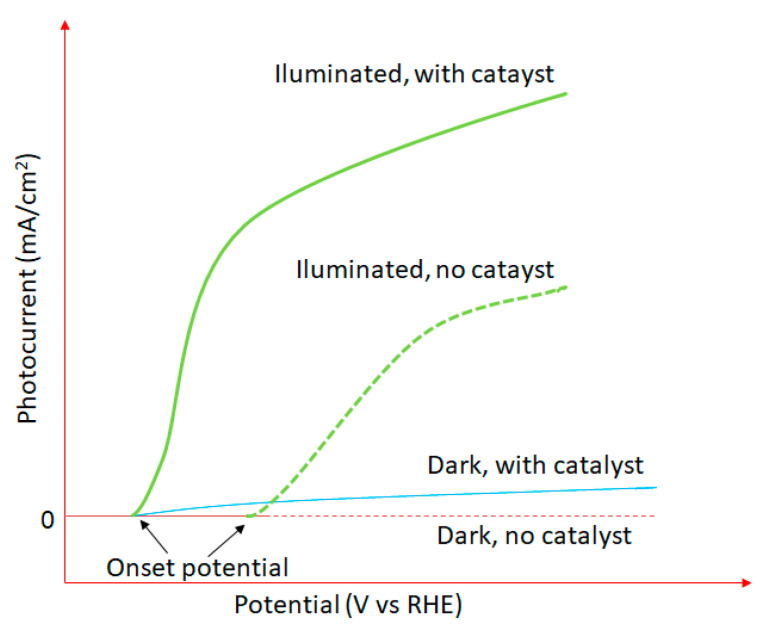
J vs. V curves for a photoanode in dark and illumination in the presence/absence of a photocatalyst. Adapted from reference [175].

In order to evaluate the performance and efficiency of materials and devices used in PEC systems, it is required to use benchmark metrics of assessment. One of the most important methods for the performance quantification of PEC systems is a photocurrent density–voltage (J–V) curve. This technique implies the measurement of the photocurrent density (mA/cm^2^) as a function of the applied voltage (V) under chopped or continuous irradiation. The presence of the anodic photocurrent is correlated to the O_2_ generation, while the cathodic photocurrent with H_2_ generation, respectively. Figure 13 displays the J–V curves for a photoanode in dark with/without catalyst and under illumination with/without catalyst [175]. The onset potential is defined as the applied potential where the current starts to increase; a lower onset potential is desired from an economical energy consumption point of view.

Alternatively, a direct correlation between solar energy and hydrogen evolution is necessary to calculate the solar to hydrogen (STH) efficiency. In order to determine this efficiency, it is necessary to quantitatively measure the produced H_2_. STH efficiency gives the overall reaction efficiency of a PEC system used for water splitting reaction under simulated solar illumination and with no external voltage applied. Also, it can be calculated only if the electrodes are immersed in the same electrolyte without other sacrificial reagents [176]. STH efficiency can be calculated using the following formula:

(5) $$\mathrm{STH}=\left(\frac{v_{H^{2}}\left(\frac{\mathrm{mmol}}{s}\right)\times 237,000\left(\frac{\mathrm{mj}}{\mathrm{mmol}}\right)}{P_{\mathrm{light}}\left(\frac{\mathrm{mW}}{{\mathrm{cm}}^{2}}\right)\times A\left({\mathrm{cm}}^{2}\right)}\right)\mathrm{AM}1.5G$$

where, ν_H2_ is the number of mmol of photogenerated H_2_, 237,000 mJ/mmol represent the Gibbs free energy, P_light_ is the power density of the incident light and A is the illuminated area of the electrode (area of the spot light). AM 1.5G (air mass 1.5 G) represents the standard reference solar spectra used to calculate the conversion efficiency of solar energy to electrical or chemical energy. It defines the effect produced by the atmosphere of the Earth on the solar radiation [177].

Also, STH efficiency can be directly calculated from the photocurrent density when the measurement of the H_2_ amount is not possible, using the formula [178]:

(6) $$\mathrm{STH}=\left(\frac{J_{\mathrm{ph}}\left(\frac{\mathrm{mA}}{{\mathrm{cm}}^{2}}\right)\times V_{\mathrm{redox}}\times \eta_{f}}{P_{\mathrm{light}}\left(\frac{\mathrm{mW}}{{\mathrm{cm}}^{2}}\right)}\right)\mathrm{AM}1.5G$$

where, J_ph_ is the photogenerated current density, V_redox_ is the redox potential of interest (1.23 V) and η_f_ is the faradaic efficiency of the hydrogen evolution reaction.

For the determination of the faradaic efficiency of hydrogen, it is crucial to know the active surface area of the catalyst (photoelectrode) and the current density passing between the two electrodes [178].

For the system where an external bias is applied, other tools are required for the calculation of the efficiency. One of them is the applied bias photon-to-current conversion efficiency (ABPE) calculated with the following formula:

(7) $$\mathrm{ABPE}=\left(\frac{J_{\mathrm{ph}}\left(\frac{\mathrm{mA}}{{\mathrm{cm}}^{2}}\right)\times V_{\mathrm{redox}}-V_{\mathrm{applied}}\times \eta_{f}}{P_{\mathrm{light}}\left(\frac{\mathrm{mW}}{{\mathrm{cm}}^{2}}\right)}\right)\mathrm{AM}1.5G$$

where V_applied_ is the potential applied between the working and counter electrodes.

This formula can be applied for PEC devices working in a three-electrode system which implies the addition of a reference electrode. As in the case of STH efficiency, for the calculation of ABPE efficiency the utilization of sacrificial reagents and chemical bias should be avoided [176].

In addition, the incident photon-to-current efficiency (IPCE) is one of the most important tools for the evaluation of PEC performances. It offers the possibility to determine the practical efficiency limits of a material that can be used in PEC systems. It can be determined using the following formula [176]:

(8) $$\mathrm{IPCE}=\left(\frac{J_{\mathrm{ph}}\left(\frac{\mathrm{mA}}{{\mathrm{cm}}^{2}}\right)\times 1239.8\left(V\times \mathrm{nm}\right)}{P_{\mathrm{mono}}\left(\frac{\mathrm{mW}}{{\mathrm{cm}}^{2}}\right)\times \lambda \left(nm\right)}\right)$$

where 1239.8 (V × nm) represents a multiplication of h (Planck’s constant) and c (the speed of light), P_mono_ is the power density of the monochromated light used for photoelectrode irradiation and *λ* is the wavelength of the light.

In 1972, Fujishima et al. reported the first photoelectrochemical water splitting reaction with solar hydrogen production opening new opportunities in the PEC field [179]. The authors used n-type TiO_2_ as photoanode and an irradiation source emitting in the ultraviolet (UV) region. Since Fujishima and Honda discovered this effect, TiO_2_ has been extensively used as photoelectrode because it is cheap, non-toxic and possesses high photostability. However, it has a wide band gap (ca. 3.2 eV; λ ≈ 396 nm) and it can absorb only the UV light which represents a small part of solar light (3–5%) [180,181].

Taking into account the excellent efficiencies of HOIPs in the photovoltaic applications, they seem to be possible candidates for integration in PEC devices. In a PEC system, the photoelectrodes must be in direct contact with an aqueous solution of electrolyte in order for the chemical reaction to take place. As already mentioned, the organic perovskites are very unstable in aqueous media, hence the presence of moisture has crucially affected their application in photoelectrochemistry [182,183]. However, many researchers have tried to overcome this drawback in order to generate a device showing excellent stability and high efficiency for PEC water splitting applications. The passivation of CH_3_NH_3_PbI_3_-based photoanode with a very thin Ni layer for both waterproof coating and the holes transfer has been reported. The device activity was almost entirely lost in an electrolyte solution 0.1 M Na_2_S (pH 12.8) after 20 min [184]. The stability was increased up to 30 min when a carbon nanotube/polymer composite layer was used as waterproof coverage layer [185]. Also, Wang et al. reported an innovative functionalization of CH_3_NH_3_PbI_3_-based photoanode with Ni as protective layer against humidity showing 30 min stability and 2.08 mA/cm^2^ value of the photogenerated current [186]. All these photoelectrodes are based on the planar perovskite solar cells, including expensive Au electrodes and hole transporting materials, making the final device very costly and irrelevant due to its unsatisfactory stability [187].

Inorganic perovskites having the ABO_3_ formula are of great interest for H_2_ and O_2_ production due to their exceptional stability and high photocatalytic activity. In Table 8 a series of different perovskites used as photoelectrodes for H_2_ and O_2_ generation via a photoelectrochemical water splitting reaction are presented.

Titanates were tested in photoelectrochemical water splitting in both acidic and basic environments. For example, BaTiO_3_ with a large band gap value (3.11 eV) shows a photocurrent density of 0.007 mA/cm^2^ at 0.5 V vs. SCE (saturated calomel electrode). The SCE electrode is a reference electrode based on mercury and mercury chloride [188]. The addition of PbTiO_3_ to TiO_2_ leads to a device with enhanced photoelectrochemical response (0.3 mA/cm^2^) compared to pure TiO_2_ (almost 0 mA/cm^2^) under visible light irradiation. The observed difference in the photocurrent density indicates that PbTiO_3_ acts as a visible light absorber [189]. Excellent photoelectrochemical activities are reported for SrTiO_3_, CaTiO_3_, LaNiO_3_ and NaTaO_3_ under UV radiation, but the utilization of visible light is restricted by their wide band gap values [190,191,192,193,194,195,196]. Therefore, perovskites having smaller band gap values are of interest in the photocatalytic field for enhanced water-splitting efficiencies. Ferrite perovskites are a class of materials which generally show the band gap energies in the visible region. The rhodamine B was successfully reduced under visible irradiation on YFeO_3_ that showed a much higher photocatalytic activity compared to TiO_2_ which possess a low absorption under visible light [197], and it was successfully tested in the form of both nanoparticles and compact films in photoelectrochemical production of H_2_ [198].

In particular, BiFeO_3_ (BFO) and LaFeO_3_ (LFO) were extensively utilized as photoelectrodes in photoelectrochemical applications, mostly for water photolysis and dye photodegradation. The dyes photodecomposition process will be detailed in the next section of this review. BFO is one of the few multiferroic materials at room temperature with a band gap value of ca. 2.7 eV and an excellent photocatalytic activity [199]. The ferroelectric properties of BFO are able to improve the electron-hole separation and, hence, the photocatalytic activity. The effect of spontaneous polarization on the charge carriers separation is extensively detailed in literature [200]. Both undoped and doped BFO prepared by PLD were tested in photoelectrochemical applications as shown in Table 8. In 0.2 M Na_2_SO_4_ electrolyte under sunlight illumination, the BFO photoanode generates a photocurrent density of 0.12 μA/cm^2^ at 0.8 V vs. Ag/AgCl. After the material was heated in a reductive atmosphere of hydrogen (5%), the photocurrent increases ca. 6 times. The difference is due to smaller recombination and more efficient separation of free carriers on the hydrogen-treated sample [201]. A value of 17% for incident photon-to-current efficiency was reported for BFO photoelectrodes prepared by chemical vapour deposition (CVD). The modification of BFO surface with Ni improves the reaction kinetics and decreases the overpotential for water oxidation. The photocurrent density increases from 0.17 to 0.72 mA/cm^2^ with Ni addition [202]. Nanowires as well as nanocubes of BFO generate only O_2_ when irradiated with UV light. A mixture of BFO and SrTiO_3_ (STO) can lead to H_2_ formation under visible light irradiation [203,204]. Also, in the case of polycrystalline BFO films, it was reported that by increasing the film crystallinity, the PEC response is enhanced. Moreover, the PEC response is strongly dependent on the spontaneous polarisation of BFO polycrystalline films [205,206]. Similarly, Cho et al. have reported epitaxial thin films of BFO deposited by PLD on STO showing excellent PEC properties [207]. Ti-doped BFO was reported to generate 0.04 mA/cm^2^ at 1.23 V vs. RHE (reversible hydrogen electrode) in a basic solution of NaOH under UV irradiation [208]. The RHE is a reference electrode with the potential independent on the pH changes. The onset potential was ca. 0.81 V vs. RHE [208]. A detailed study on Y-doped BFO used as photoanode with high PEC efficiency for water splitting reaction (J_ph_ = 0.8 mA/cm^2^ at 1.4 V vs. RHE) was recently reported in literature [209]. Moreover, complex BFO-heterostructures were used as photoelectrodes for water splitting reaction. For example, nanofibers of BiFeO_3_/Bi_2_Fe_4_O_9_ with enhanced stability show higher efficiency for H_2_ production compared to pure BFO and Bi_2_Fe_4_O_9_. This improved efficiency was attributed to the formation of well-defined heterojunctions between the component materials, which helps the separation of charge carriers and avoids their recombination [210]. A significant increase in the photocurrent density compared to pure BFO was also observed for BiFeO_3_/Fe_2_O_3_. The heterostructure having a concentration of 9% Fe_2_O_3_ shows a J_ph_ value of ca. 0.19 mA/cm^2^ (at 0.6 V vs. Ag/AgCl), using visible irradiation light, which is about three times higher than that obtained for pure BFO (0.055 mA/cm^2^) [211]. Recently, a complex device based on WO_3_/BiVO_4_/BiFeO_3_/FTO (fluorine-doped tin oxide) was reported to show a huge value of the photocurrent density under solar irradiation in 0.5 M Na_2_SO_4_ electrolyte (46.9 mA/cm^2^ at 2.53 V vs. RHE) [212]. The great response is correlated to the great separation of photogenerated charge carriers due to the similar band alignment of the three component materials deposited on the FTO substrate. The presence of an internal electric field at the BiVO_3_/BFO p-n junction interface together with BFO polarization, which improve the electron-hole pairs separation, have also been noticed [212].

LaFeO_3_ is a perovskite with a smaller band gap value than BiFeO_3_, more specifically ca. 2.07 eV, offering the possibility for a better absorption in the visible range [180,213]. Several years ago, Celorrio et al. studied the photoelectrochemical properties of nanostructured LFO photocathode manufactured by the screen-printed technique. They reported higher cathodic than anodic photocurrent values [214]. In screen-printed technology, the perovskite material is combined to an organic compound which is used to improve the adhesion to the substrate. The mixture is printed to the substrate using a heater, followed by calcination [215]. Also, in another study, the simultaneous generation of O_2_ and H_2_ using LFO powders was noted [216]. The values of the photogenerated current are around 0.8 mA/cm^2^ at potentials higher than 1 V vs. Ag/AgCl for utilisation of LFO as photoanode under simulated solar light [217]. Yu and coll. have deposited p-LFO and p-LFO/n-Fe_2_O_3_ on ITO (indium tin oxide) conductive substrates by PLD. The fabricated photocathodes show enhanced stability in alkaline NaOH electrolyte solution under visible irradiation even after 120 h [218]. May et al. [219] have reported an accurate study on the effect of thickness of LaFeO_3_/Nb:SrTiO_3_ (LFO/STON) ultrathin films prepared by PLD on the photoelectrochemical properties. The cathodic photocurrent was shown to be strongly dependent on the film thickness, being observed only for samples with 10–25 nm thickness. The anodic photocurrent is observed for both thinner and thicker than 10 nm LFO films [219]. A photoanode based on Cu-doped LFO was reported to show a photocurrent density of 2 mA/cm^2^ at 1.1 V vs. Ag/AgCl under visible irradiation [220]. Notably, no reports on utilization of devices based on heterostructures of LFO/BFO perovskites for water splitting reaction have been published to date.

**Figure 14:**
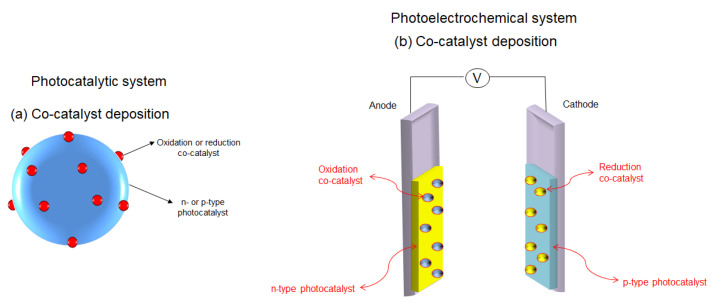
The schematically representation of (**a**) photocatalyst; (**b**) photoelectrode loaded by co-catalysts. Adapted from reference [168].

The utilization of co-catalysts can further improve the global efficiency of water splitting reaction. Generally, noble metals (Pt, Rh, Pd) and metal oxides (NiO, RuO_2_) are extensively used as co-catalysts. When a photoelectrode is loaded with a co-catalyst, the photogenerated electrons which arrive at the surface are entrapped by the co-catalyst. In this way, the utilisation of co-catalysts, stimulates the surface chemical reaction and constrains the backward reaction, leading to an increased overall water splitting efficiency [90]. A schematic representation of a photocatalyst and a photoelectrode loaded by co-catalysts is presented in Figure 14.

**Figure 15:**
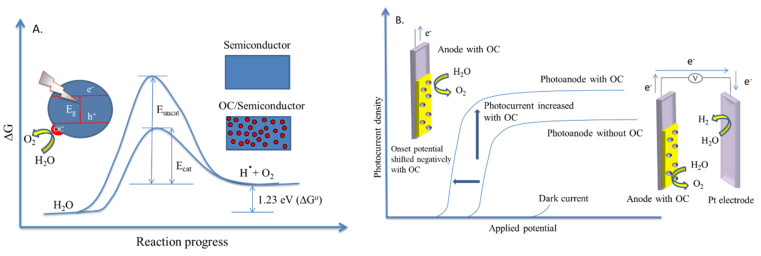
The working principle of the reaction in the presence of photocatalysts (**A**) and photocurrent density vs. the applied potential curves of photoelectrodes (**B**) loaded with an oxidation co-catalyst for both dark and illuminated processes, where OC—oxidation cocatalyst, E_uncat_—activation energy without OC and E_cat_—activation energy with OC. Adapted from references [168,228].

Also, the working principle of the reaction in the presence of photocatalysts (a) and photoelectrodes (b) loaded with an oxidation co-catalyst for both dark and illuminated processes is illustrated in Figure 15.

Polycrystalline films of BFO synthesized via the citrate method, decorated with nanoparticles of Ag which act as co-catalysts, were reported. This heterostructure shows almost two times higher photocurrent density compared to pure BFO [229]. Also, a device based on reduced graphene oxide (rGO)/BFO was reported to ensure an excellent charge carriers separation, leading in this way to a higher IPCE [230]. Moreover, thin films of BiFeO_3_ show water-splitting activity without any external bias applied [200,221].

#### 4.1.2. Catalytic Combustion of Methane

Natural gas (a hydrocarbon gas mixture consisting mainly of methane) has a major impact on our lives, being one of the most used energy sources. Thermal combustion of light hydrocarbons meets many challenges because of its low efficiency and high pollution degree. This conventional combustion process takes place at high temperatures (above 1300 °C) generating high amounts of nitrogen oxides (NO_x_), which severely affect the environment and, furthermore, human health. In the last few years, the market request has shown a significant increase of fuel consumption, leading, at the same time, to an advanced pollution risk. The catalytic combustion is preferred instead of the flame combustion due to its major advantages, such as enhanced fuel efficiency and lower emission of pollutants in the exhaust gas [3,5]. Important quantities of methane (CH_4_) are annually consumed in the fuel industry, it being considered one of the cleanest sources of fossil energy [231]. The simplified chemical reaction of methane combustion is:

CH_4_ + 2 O_2_ → CO_2_ + 2 H_2_O (9)

However, beside the production of CO_2_ and H_2_O, during the methane combustion process, many other reactions can take place, such as partial combustion of methane with CO formation, steam reforming and others [232]:

CH_4_ + 3/2 O_2_ ⇒ CO + 2 H_2_O (10)

CH_4_ + H_2_O ⇄ CO + 3 H_2_ (11)

2 H_2_ + O_2_ ⇒ 2 H_2_O (12)

CO + H_2_O ⇄ CO_2_ + H_2_ (13)

2 CO + O_2_ ⇒ 2 CO_2_ (14)

In contrast to the classical combustion, the heterogeneous catalytic combustion of methane operates at lower temperatures (<800 °C), increases the conversion efficiency of methane into energy, and severely decreases the atmospheric pollutants emission [233]. It is worth noting that catalytic combustion of methane is important not only for power generation but, also, for air pollution abatement [234]. The ideal catalyst for methane combustion is required to possess high thermal and chemical stability, but at the same time it should present catalytic oxidation activity in the low-temperature range [4]. The most promising low-temperature catalysts for this reaction are the supported noble metals. Excellent catalytic activities for methane combustion are reported on supported platinum [235], rhodium [236] and palladium [237]. Unfortunately, they are expensive and in a strong oxidative atmosphere they are susceptible to form volatile oxides leading to a decrease of the catalytic activity. Also, at high temperatures, the noble metal particles tend to accumulate and to form a compact material (the so-called sintering effect). This effect decreases the specific surface area (SSA) of the catalyst and, implicitly, its catalytic activity [4]. These disadvantages made researchers look for a possible replacement of this kind of catalysts. A good alternative is to use active catalysts for hydrocarbons combustion based on single or binary oxides of transition metals. A few single oxides active for hydrocarbons combustion are Co_3_O_4_, CuO, NiO, MnO_2_. The combination of oxides generally gives a greater thermal stability and higher combustion activity compared to the single oxides [45].

Inorganic perovskites ABO_3_ have been found to be adequate catalysts for total oxidation since they combine the high catalytic activity with the low volatility [3]. For the perovskite-type oxide catalysts, two types of oxygen species having different bonding strength are present: (i) adsorbed on the surface and (ii) lattice oxygen. It is believed that the adsorbed oxygen is more active, reacting with methane at lower temperatures than the lattice oxygen. At higher temperatures, the coverage of the adsorbed oxygen decreases, while the lattice oxygen becomes highly active. Voorhoeve et al. have proposed two reaction types for catalytic combustion of methane over perovskite materials: suprafacial and intrafacial. The suprafacial reaction is mainly present at low temperature, while with increasing the temperature, the intrafacial process occurs [45].

Table 9 presents the performances of different undoped perovskites used as catalysts for methane combustion. Lanthanum cobaltate (LaCoO_3_), having a SSA smaller than 15 m^2^/g. shows T_50_ (temperature corresponding to 50% methane conversion) values ranging between 525 and 709 °C [45,50,56,57,58,59,63,64] depending on the reaction conditions, i.e., methane and oxygen concentrations in the feed gas and the magnitude of the gas flow rate. With the increase of the SSA, T_50_ decreases, reaching a minimum temperature of 449 °C for LaCoO_3_ with an SSA of 43 m^2^/g [65]. This temperature can be further lowered by adding noble metals (e.g., Pt, Pd) to the LaCoO_3_ perovskite structure [61]. The replacement of La^3+^ with Pr^3+^ leads to a major increase of T_50_ and of the activation energy (E_a_), which is, in fact, the required energy for a chemical reaction to take place. This behavior is correlated to the redox properties of Pr^4+^/Pr^3+^ couple which can affect the valence state of cobalt ions during methane combustion reaction [56]. Lanthanum manganate (LaMnO_3_) shows excellent catalytic activity for the methane combustion with T_50_ in the 500–600 °C interval [50,55,57,64,70,72,75,80,82,238]. However, the value of T_50_ can be further decreased by changing the synthesis method of LaMnO_3_ perovskites. For example, LaMnO_3_ prepared by the flame-hydrolysis method shows a T_50_ of 489 °C, while that prepared by the citric acid and flame pyrolysis methods show T_50_ values of 446 and 435 °C, respectively [61,65,66]. No major changes in the catalytic activity were observed when La^3+^ was replaced by Pr^3+^ or Gd^3+^ [69]. However, it severely decreases for NdMnO_3_ and SmMnO_3_ because of their easier reductibility [72]. It is worth noting that, due to its good thermal stability and high activity, which are further improved after dispersion on a lanthanum-stabilized alumina-coated monolith [70], LaMnO_3_ is an active phase of choice in high-pressure structured catalytic combustors [239,240,241,242,243]. Lanthanum ferrite (LaFeO_3_), which shows the highest stability compared to LaCoO_3_, LaNiO_3_ and LaMnO_3_, displays a catalytic activity similar to LaMnO_3_ [3,45,57,61,63,64,65,69,76,77,78,244]. Ciambeli et al. have reported a detailed study on ferrite perovskites (AFeO_3_) containing different rare earth cations (La^3+^, Sm^3+^, Nd^3+^) in A sites. It was demonstrated that LaFeO_3_ catalyst shows the best catalytic activity for methane combustion, with total selectivity to CO_2_, having the T_50_ = 528 °C and the temperature corresponding to 90% methane conversion (T_90_) of ca. 615 °C [77]. Spinicci et al. have revealed that La_0.9_FeO_2.85_ shows better activity compared to stoichiometric LaFeO_3_. The further decrease of lanthanum and oxygen contents leads to the loss of the catalytic activity [245]. The catalytic activity can be limited by the low specific surface area of the perovskite catalysts [45]. One of the most used methods to increase the SSA of catalysts based on perovskites is to disperse them on appropriate supports [246]. Indeed, it has been shown that the SSA of the unsupported BaTiO_3_ and PbTiO_3_ increased from 0.4 and 0.5 m^2^/g, respectively, to 193 and 175 m^2^/g for their alumina-supported counterparts, respectively. The increase of the SSA led to an enhanced catalytic activity for methane combustion [44,247].

The catalytic activity of the perovskites is strongly dependent on B-site cations, while A-site cations are responsible for structure stability, as already mentioned in Section 2.1. Therefore, by substituting small portions of A and/or B cations with other transition metals, both the catalytic activity and thermal/chemical stability of the final catalyst can be improved. Generally, the most used A-site dopants for perovskites are alkaline earth metals (Sr, Ca and Ba) [3,45,53,58,61,71,73,74,76,78,79] and lanthanides (Ce, Eu) [45,51,58,61,71], as can be observed in Table 10 where the performance of A-site doped perovskites in methane combustion is resumed. Arai et al. [45] reported that La_0.6_Sr_0.4_MnO_3_ perovskite prepared by solid state reaction exhibits similar catalytic activity to Pt/Al_2_O_3_ at low temperatures (350–550 °C). At temperatures higher than 600 °C it becomes less active than the noble metal catalyst. However, La_0.6_Sr_0.4_MnO_3_ was shown to be the most active catalyst compared to Ca-doped LaMnO_3_, Sr-doped LaFeO_3_, Ba-doped LaCoO_3_, Ce-doped LaCoO_3_ and Ca-doped LaCoO_3_ [45]. The high activity of La_0.6_Sr_0.4_MnO_3_ for methane combustion at low temperatures was confirmed in other studies [71,74]. This behavior was correlated with its high capability to adsorb oxygen on the surface, most probably due to the fact that the presence of the bivalent dopant determines the formation of Mn^4+^ species, [45,71,74]. When the temperature increases, the amount of adsorbed oxygen decreases and, hence, its catalytic activity. Calcium is the most used element for LaFeO_3_ doping, as can be seen in Table 10. Pecchi et al. [76] reported a detailed study concerning La_1-x_Ca_x_FeO_3_ perovskites prepared by citrate and co-precipitation methods. For the samples prepared by co-precipitation, there are almost no changes in T_50_ with the calcium content, while for those prepared via the citrate method the addition of calcium minimizes T_50_ [76]. Similar results on Ca-doped LaFeO_3_ prepared by the citrate method were also reported elsewhere [79].

In Table 11 the performances of B-doped perovskites in the catalytic combustion of methane are presented. Saracco et al. studied LaCr_1-x_Mg_x_O_3_ perovskite prepared by the citrate method and observed that its catalytic activity increases with the Mg content [47]. The opposite behaviour was observed for LaAl_1-x_Mn_x_O_3_, whose catalytic activity decreases with the Al content. LaAl_0.2_Mn_0.8_O_3_ shows the best activity, while the activity of LaAl_0.4_Mn_0.6_O_3_ is similar to the pure LaMnO_3_ [55]. A high fraction of Fe^4+^ is observed in LaFe_1-x_Mg_x_O_3_ perovskite and it increases with the addition of Mg content leading to lower catalytic activity at low temperatures [77]. Taguchi et al. [248] reported that Ca(Mn_0.6_Ti_0.4_)O_3_ shows better catalytic activity compared to undoped LaFeO_3_ and (La_0.8_Sr_0.2_)(Cu_0.15_Fe_0.85_)O_3_ perovskites. In the case of Ca(Mn_0.6_Ti_0.4_)O_3_ catalyst, the T_50_ value is ca. 580 °C, while for LaFeO_3_ and (La_0.8_Sr_0.2_)(Cu_0.15_Fe_0.85_)O_3_ it increases up to 800 and 780 °C, respectively. The significantly lower T_50_ value of the Ca(Mn_0.6_Ti_0.4_)O_3_ system was attributed to the increased content of Mn^3+^ cations which act as oxygen adsorption sites [78,248,249]. Moreover, even a lower T_50_ value was reported for Al-doped LaMnO_3_. Thus, for 10% Al^3+^ content, the T_50_ is ca. 520 °C and increases with the Al content. Notably, the T_50_ value decreases with the increase of the SSA, the highest SSA (22 m^2^/g) corresponding to the La(Mn_0.9_Al_0.1_)O_3_ system [250]. Recently, Miao et al. [251] reported a catalyst based on La(Mn,Fe)O_3_ with an excellent stability at 550 °C in the catalytic combustion of methane. The methane conversion was almost entirely preserved after eight combustion cycles [251].

### 4.2. Applications of Perovskite-Type Materials in the Removal of Pollutants from Waste Waters

In the last few years, the high extent of pollution has become one of the biggest problems facing humanity because it can severely affect human life [252]. The residual dyes emerging from different industries (pharmaceutical, textile, paper and others) are considered to be the most common water pollutants. For example, in the textile industry, every kilogram of final product generates 50–100 L of waste water [253,254,255]. Numerous techniques for water treatment have already been developed, such as adsorption [256], chemical coagulation [257], electrocoagulation [258], advanced oxidation processes [259], photocatalysis [260] and photoelectrocatalysis [261]. The adsorption method used in waste water treatments involves the adhesion of pollutants (organic dyes, in this case) to the surface of a solid material, called adsorbent. The adsorbent properties of solid materials can be explained by the force fields which govern the surface properties. Depending on the type of forces established between the adsorbent and the adsorbed molecules, the adsorption can be: physical adsorption (for weak forces) and chemisorption (chemical bonds). The main characteristics controlling the adsorption efficiency of an adsorbent are the specific surface area and porosity. The adsorption method has the advantage that it can be used for a large amount of water with low pollutants content using simple and relatively cheap operating systems [262]. The most commonly used adsorbents for the elimination of organic dyes from water are: activated carbon [263,264], alumina [265,266], bentonite [267,268] and zeolites [269]. The main drawbacks of these techniques are correlated to the adsorbents’ capacity which decreases in time and the high costs of their regeneration. Moreover, depending on the pollutant’s nature, the adsorbent could be irreversibly blocked [262]. Chemical coagulation is an extensively used method for the waste water treatment. It is based on the utilization of coagulants which have the ability to form precipitates. The pollutants are trapped in the formed precipitate which is settled, offering the possibility to easily separate the supernatant (water) from sediments. The process is strongly dependent on the pH, coagulant concentration and mixing. Depending on the nature of the pollutant, the coagulant can be either inorganic (AlCl_3_; FeCl_3_) or organic (poly diallyldimethyl-ammonium chloride; polyacrylamide). This method is able to remove high amounts of organic dye from water, but it leads to an increased cost of the process due to two drawbacks: (i) it requires high quantities of chemical coagulant, and (ii) it produces large amounts of sludge [270,271]. The electrocoagulation is similar to the chemical coagulation, in this case the coagulants being electrochemically generated. It uses low-voltage and two sacrificial iron (or aluminum) electrodes. Under an applied current voltage, at anode are generated Fe^3+^ (or Al^3+^) species, while at cathode water is reduced to H_2_ and hydroxides. The ions formed at the anode surface react with hydroxide groups, leading to the coagulants’ production. The resulting quantity of sludge is much lower compared to the chemical coagulation [270,272]. However, it requires often the replacement of the sacrificial electrodes to preserve a high efficiency. The operating cost of this technique increases significantly when applied in non-electrified areas, this being a common case for waste water found in nature [273]. One of the most used advanced oxidation methods for the degradation of dyes from waste water is ozonation. Ozone has a very high oxidation potential being capable to oxidize the organic dyes [259]. Unfortunately, ozone is very toxic for the human body, its strong oxidizing character can cause various diseases [274]. The photocatalytic and photoelectrochemical processes are considered to be the key solution to remove environmental pollution because they are clean methods which use renewable solar energy, their advantages have been already discussed in this review.

Rhodamine B is an important cationic xanthene dye being one of the mostly used model organic dye in photodegradation studies. Important quantities of this organic dye are coming from the textile industry contributing to environmental pollution [275,276,277,278]. Equally, methyl orange (anionic dye) and methylene blue (cationic dye) are considered harmful dyes released from textiles and printing industries and it is desired to minimise as much as possible their concentration in the environment [279,280,281,282,283,284]. Besides these, other common organic pollutants targeted in photodegradation applications are congo red (highly toxic and carcinogenic pollutant) [285], neutral red [286], phenol red [287], 4-methylphenol [288] and tetracycline [289], their chemical structures being presented in Table 12.

The photooxidation reaction of organic dyes is similar to the processes involved in the water-splitting reaction. When the semiconductor is irradiated, the photons having energies higher than its band gap energy are absorbed. The photogenerated charge carriers migrate to the photocatalyst surface. Once there, electrons react with adsorbed oxygen molecules (O_2(ads)_) generating initially superoxide radicals (O_2_^•^) and, then, hydroperoxide radicals (HOO^•^). On the other hand, the positive holes interact with the surface-adsorbed water molecules (H_2_O_(ads)_) producing free hydroxyl radicals (OH^•^). When the organic compounds (dyes) are adsorbed on the photocatalyst surface, they are rapidly oxidized by the highly reactive radicals to CO_2_ and H_2_O [296]. The photodegradation of organic compounds can be schematically presented by the following series of reactions, where P = photocatalyst:

P + hν → P(h^+^_VB_) + P(e^−^_CB_) (15)

P(h^+^_VB_) + H_2_O_(ads)_ → P + OH^•^ + H^+^ (16)

OH^•^ + organic dye → CO_2_ + H_2_O (17)

P(e^−^_CB_) + O_2(ads)_ → P + O_2_^•^ (18)

O_2_^•^ + organic dye → CO_2_ + H_2_O (19)

As in the case of hydrogen production from water, TiO_2_ is the most utilized photocatalyst for water depollution. It was used as photocatalyst for organic dye photooxidation in both powder and immobilized form, with good photocatalytic activity [297,298,299]. Its activity was further improved when an external bias was applied to the system. However, pure TiO_2_ has high rate of the charge carrier’s recombination and wide band gap value, which are the main drawbacks of this semiconductor in photocatalysis [164,300].

Structural and compositional properties of oxide perovskites are suitable for the photodegradation of organic molecules, their performances being summarised in Table 13. SrTiO_3_ shows excellent photocatalytic activity under UV irradiation for the degradation of rhodamine B [301,302] and methyl orange [303]. The rhodamine B was completely degraded after 1.3 h, while 3 h were necessary to completely eliminate methyl orange dye. Under visible irradiation, the degradation efficiency of SrTiO_3_ is lower than 50%, because of its large band gap [304].

The doping of SrTiO_3_ with different elements (Fe, Nb) leads to an improvement of the photocatalytic efficiency under visible light irradiation [289,304,305]. LaCoO_3_ shows even higher photocatalytic activity under UV irradiation for the degradation of rhodamine B, with a complete photooxidation of the organic dye after 0.8 h [305]. Its activity considerably decreases under visible irradiation [306].

The degradation of methyl orange was performed with conversions higher than 90% over BiFeO_3_ photocatlaysts under both UV and visible light [307]. Moreover, nanoparticles of BiFeO_3_ and Gd-doped BiFeO_3_ (Bi_1-x_Gd_x_O_3_, where *x* = 0, 0.05, 0.1, 0.15) prepared by classical citrate method were tested in the photodegradation of MB. It was observed that while increasing the Gd content, the photocatalytic activity increases as well, reaching the highest conversion of the organic dye (94%) with Bi_0.9_Gd_0.1_FeO_3_. The increased ferromagnetic nature of Bi_0.9_Gd_0.1_FeO_3_ is considered responsible for its different photocatalytic behavior in MB degradation [308]. A similar degradation efficiency (94%) was observed for rhodamine B over Gd-doped BiFeO_3_ [309]. Congo red dye was almost completely oxidized on Ca-doped BiFeO_3_ ultrafine nanofibres (Ca_x_Bi_1-x_FeO_3_, where *x* = 0, 0.05, 0.1, 0.15) and the photocatalytic conversion of the dye increased along with the Ca content [310]. La_x_Bi_1-x_FeO_3_ (*x* = 0, 0.05, 0.1, 0.15) perovskites were used as photocatalysts for the photooxidation of the same dye, almost 80% of it being oxidized on La_0.1_Bi_0.9_FeO_3_ after 3 h. Further increasing the La content decreases the conversion to ca. 50% [311].

A heterostructure based on BiFeO_3_/CuWO_4_ was tested for the degradation of methyl orange (MO) and rhodamine B (Rh B). The dyes’ concentrations were spectrometrically analyzed in time, and the absorption peaks of MO and Rh B disappeared almost entirely after 120 and 75 min, respectively. The higher photocatalytic activity observed on BiFeO_3_/CuWO_4_ compared to single components was correlated with the heterojunction formation between the two oxides. The most beneficial BiFeO_3_:CuWO_4_ ratio for the organic dyes photodegradation was found to be 1:1 [312]. Methyl violet (MV) was photooxidized up to 93% on a heterostructure based on p-n heterojunction BiFeO_3_/TiO_2_ under visible irradiation as an effect of the poor recombination of charge carriers and strong absorption properties of the device [313].

Great efficiencies were reported for pure LaFeO_3_ used as photocatalyst under visible irradiation for the degradation of 4-methylphenol [314], rhodamine B [315,316], and methyl orange [317]. Nanocubes, nanospheres and nanorods of LaFeO_3_ were used as photocatalysts for Rh B oxidation under visible irradiation. No remarkable differences were observed between the nanorod and nanosphere morphologies, the organic dye conversions being similar. On the other hand, for the nanocube morphology the conversion efficiency was much lower. Irrespective of their morphology, all LFO samples showed better activities than Degusa P25 TiO_2_.

The higher efficiency of LFO is explained by its strong absorption properties in the visible region. Furthermore, the presence of an enhanced density of adsorbed oxygen or oxygen from surface hydroxyl groups on LFO photocatlaysts, which can act as efficient oxidizing agent, was confirmed by X-ray photoelectron spectroscopy (XPS) [318]. Wei et al. [319] reported that the doping of LaFeO_3_ with Mn ions leads to a perovskite structure of LaFe_0.5_Mn_0.5_O_3_ having higher oxygen vacancies and excellent absorption properties for visible light. All these, together with the variable valency of Mn cations contribute to the generation of a photocatalyst with improved activity for photodegradation of MO. It was also noted that the reaction mechanism is pH-dependent: the global yield decreased with the increasing of the pH value [319]. Recently, numerous scientific papers based on a heterostructure of nanosheets of graphitic carbon nitride (g-C_3_N_4_)/LFO nanoparticles were reported. High values of the photogenerated current were obtained on 15% g-C_3_N_4_/LFO (~9 μA/cm^2^) and 20% g-C_3_N_4_/LFO (~17 μA/cm^2^) compared to pristine LFO (0.04 μA/cm^2^). Rh B was almost completely oxidized on g-C_3_N_4_/LFO after ca. 2 h under visible light irradiation. Moreover, the photocatalytic device showed excellent stability, its activity being entirely maintained after 4 catalytic cycles [320,321]. This photocatalyst was also used with good results for Brilliant Blue (BB) degradation [322].

## 5. Conclusions and Perspectives

The properties and (photo)catalytic behavior of perovskite materials in water splitting, catalytic combustion of methane and photo-degradation of pollutants from water have been presented and discussed based on more than 300 relevant papers, leading to the following conclusions:

➢ There are various synthesis methods for both powder and thin films, which determine their physicochemical properties. The specific surface area of the perovskite powders, which is a key characteristic of a solid catalyst, is strongly influenced by the preparation method used, but remains low. Indeed, the highest surface areas, mainly obtained by citrate and flame-pyrolysis methods, do not exceed several tens of m^2^/g. On the other hand, pulsed laser deposition is one of the most suitable preparation methods for inorganic perovskite thin films, due to its high material transfer efficiency, precise control and the great flexibility of the process. Depending on the experimental conditions, the stoichiometry of the material, as well as the thickness and the crystallinity of the films can be controlled. The most commonly used lasers for the preparation of perovskite films are those emitting in UV spectrum (193 nm, 248 nm and 355 nm). The films’ thickness starts from less than 1 nm and rises up to ca. 600 nm.
➢ Oxide ferroelectric perovskites show excellent efficiency for the conversion of solar energy into chemical energy (H_2_) via water splitting. Both photocatalytic and photoelectrochemical systems are extensively studied in this application domain. The high spontaneous polarization of BiFeO_3_ is beneficial for a very efficient electron-hole separation. LaFeO_3_ presents strong absorption properties of visible light, which represents ca. 42% of the entire solar spectrum. The photoelectrodes are tested for a wide range of pH values, starting from semi-acidic to strong alkaline media. The highest photocurrent density (46.9 mA/cm^2^ at 2.53 V_RHE_) is obtained for a complex heterostructure based on WO_3_/BiBO_4_/BiFeO_3_. The best stability (more than 120 h) was reported in 1M NaOH for p-LaFeO_3_/n-Fe_2_O_3_.
➢ Due to their good thermal stability, perovskite materials were successfully used in the catalytic combustion of methane for both power generation and methane emission abatement. Although the performance of pure perovskites is limited by their small specific surface area, their efficiency can be improved either by dispersion onto support materials possessing high surface area and thermal stability or by doping with other transition metals. Indeed, substitution in A and B sites of the perovskite structure with small amounts of other cations can improve both the stability and activity of the catalyst. Improved activity and stability can also be obtained by coating of the supported perovskite either on ceramic or metallic monoliths. The most used A-site dopants for perovskites are alkaline earth metals (Sr, Ca and Ba) and lanthanides (Ce, Eu), while for B-sites metals from the 3 and 4 periods (Mg, Al, Mn and Cu) in particular are preferred. The most active perovskites for the low-pressure methane combustion is La_0.6_Sr_0.4_MnO_3_ with a value of T_50%_ of 360 °C. The high activity of this catalyst is due to its enhanced ability to adsorb oxygen on the surface.
➢ The photodegradation of organic dyes on inorganic semiconducting perovskites showed excellent results. Their high stability under extreme chemical conditions, strong absorption properties and efficient charge separation lead to high photocatalytic activity even after several reaction cycles. Catalytic systems containing BiFeO_3_ perovskites as such or modified with different dopants exhibited an exceptionally high activity in the photocatalytic degradation of both anionic and cationic organic dyes.

Due to their high chemical and thermal stabilities together with their large compositional flexibility, perovskites remain (photo)catalytic materials of choice for applications in energy production and environmental protection. Obviously, new preparation procedures and the improvement of existing ones will allow in the future not only higher surface areas to be obtained but also new morphologies with consequences for their (photo)catalytic behavior.

## Figures and Tables

**Table 1:** The influence of the synthesis method on the specific surface area (SSA) of different perovskite powders.

Catalyst	Preparation Method	SSA (m^2^/g)	Ref.
BaTiO_3_	Solid state reaction	0.4	[44]
PbTiO_3_		0.5	
LaAlO_3_	Citrate	4.0	[55]
LaAl_0.95_Mn_0.05_O_3_		8.0	
LaAl_0.9_Mn_0.1_O_3_		7.0	
LaAl_0.8_Mn_0.2_O_3_		25.0	
LaAl_0.6_Mn_0.4_O_3_		25.0	
LaAl_0.4_Mn_0.6_O_3_		26.0	
LaAl_0.2_Mn_0.8_O_3_		33.0	
LaCrO_3_	Solid state reaction	1.9	[45]
LaCrO_3_ and	Citrate	~5–7	[47]
LaCr_1-x_Mg_x_O_3_ with 0.1 ≤ x ≤ 0.5			
LaCr_0.5_Mg_0.5_O_3_	Citrate	6.08	[48]
LaCr_0.5_Mg_0.5_O_3_∙2MgO		13.6	
LaCr_0.5_Mg_0.5_O_3_∙6MgO		24.2	
LaCr_0.5_Mg_0.5_O_3_∙17MgO		36.9	
LaNiO_3_	Solid state reaction	4.8	[45]
LaNiO_3_	Plasmochemical	17.0	[51]
La_0.87_Sr_0.13_Mn_0.2_Ni_0.8_O_3-x_	Freeze drying	12.7	[52]
La_0.4_Sr_0.6_Mo_0.1_Ni_0.9_O_3_ (microstructured)	Freeze drying	10.3	[53]
La_0.4_Sr_0.6_Mn_0.4_Ni_0.6_O_3_ (microstructured)	Freeze drying	15.9	[53]
LaCuO_3_	Solid state reaction	0.6	[45]
LaMn_0.8_Cu_0.2_O_3_	Citrate	19.0	[50]
LaMn_0.6_Cu_0.4_O_3_	Citrate	14.0	[50]
LaFe_0.84_Cu_0.16_O_3_	Citrate	4.0	[49]
LaCo_0.8_Cu_0.2_O_3_	Citrate	21.0	[38]
LaCo_0.6_Cu_0.4_O_3_	Citrate	13.0	[38]
(Dy-Y)NiO_3_	Mechanochemical	9.5	[51]

**Table 2:** The influence of the synthesis method on the specific surface area of different powder lanthanide cobaltate-based perovskites.

Catalyst	Preparation Method	SSA (m^2^/g)	Ref.
LaCoO_3_	Co-precipitation	3.5	[56]
LaCoO_3_	Co-precipitation	8.0	[57]
LaCoO_3_	Solid state reaction	3.0	[45]
LaCoO_3_	Citrate	11.3	[58]
LaCoO_3_	Citrate	6.0	[59]
LaCoO_3_	Citrate	15.0	[50]
LaCoO_3_	Combustion	5.7	[63]
LaCoO_3_	Ultrasound spray combustion	5.5	[64]
LaCoO_3_	Flame-pyrolysis	43.0	[61]
LaCoO_3+x_	Flame-hydrolysis	15.6–22.8	[65]
La_0.8_Ca_0.2_CoO_3_	Solid state reaction	2.0	[45]
La_0.9_Sr_0.1_CoO_3_	Flame-pyrolysis	52.0	[61]
La_0.8_Sr_0.2_CoO_3_	Solid state reaction	4.7	[45]
La_0.8_Sr_0.2_CoO_3_^a^	Freeze-drying	16.5	[53]
La_0.66_Sr_0.34_CoO_3_^a^		17.4	
La_0.6_Sr_0.4_CoO_3_	Solid state reaction	3.6	[45]
La_0.8_Ba_0.2_CoO_3_		5.1	
La_0.53_Sr_0.47_Fe_0.2_Co_0.8_O_3_^a^	Freeze-drying	13.4	[53]
La_0.4_Sr_0.6_Fe_0.4_Co_0.6_O_3_^a^		10.4	
La_0.55_Sr_0.45_Mn_0.1_Ni_0.3_Co_0.6_O_3_^a^		15.0	
La_0.5_Sr_0.5_Fe_0.04_Ni_0.1_Co_0.86_O_3_^a^		19.6	
La_0.55_Sr_0.45_Fe_0.05_Ni_0.3_Co_0.65_O_3_^a^		15.2	
La_0.66_Sr_0.34_Ni_0.3_Co_0.7_O_3_^a^		18.8	
La_0.7_Sr_0.3_Ni_0.3_Co_0.7_O_3_^a^		22.7	
La_0.95_Ce_0.05_CoO_3_	Citrate	8.7	[58]
La_0.9_Ce_0.1_CoO_3_		10.0	
La_0.9_Ce_0.1_CoO_3_	Flame-pyrolysis	62.0	[61]
La_0.8_Ce_0.2_CoO_3_	Solid state reaction	3.1	[45]
La_0.8_Ce_0.2_CoO_3_	Citrate	14.2	[58]
La_0.7_Ce_0.3_CoO_3_		14.3	
La_0.6_Ce_0.4_CoO_3_		11.6	
La_0.5_Ce_0.5_CoO_3_		18.0	
PrCoO_3_	Co-precipitation	5.1	[56]
NdCoO_3_		1.6	
GdCoO_3_		2.1	

^a^ Microstructured powders.

**Table 3:** The influence of the synthesis method on the specific surface area of different lanthanide manganate-based perovskite powders.

Catalyst	Preparation Method	SSA (m^2^/g)	Ref.
LaMnO_3_	Co-precipitation	~15.0	[3]
LaMnO_3+x_	Co-precipitation	8.0	[69]
LaMnO_3_	Co-precipitation	7.0	[57]
LaMnO_3_	Co-precipitation	11.0	[70]
LaMnO_3_	Solid state reaction	4.0	[45]
LaMnO_3_	Citrate	5.6	[49]
LaMnO_3_	Citrate	20.0	[71]
LaMnO_3_	Citrate	20.0	[72]
LaMnO_3_	Citrate	22.0	[55]
LaMnO_3_	Citrate	68.0	[66]
LaMnO_3_	Citrate	22.0	[50]
LaMnO_3+x_	Flame-hydrolysis	15.6–22.8	[65]
LaMnO_3_	Flame-pyrolysis	56.0	[61]
LaMnO_3_	Ultrasound spray combustion	21.8	[64]
La_0.8_Ca_0.2_MnO_3_	Solid state reaction	6.7	[45]
La_0.9_Sr_0.1_MnO_3_	Flame-pyrolysis	51.0	[61]
La_0.8_Sr_0.2_MnO_3_	Flame-pyrolysis	70.0	[61]
La_0.8_Sr_0.2_MnO_3_	Solid state reaction	8.6	[45]
La_0.8_Sr_0.2_MnO_3+x_	Co-precipitation	5.0	[73]
La_0.6_Sr_0.4_MnO_3_	Citrate	18.7	[71]
La_0.6_Sr_0.4_MnO_3_	Solid state reaction	3.3	[45]
La_0.6_Sr_0.4_MnO_3_^a^	Citrate	4.32	[74]
SrTi_0.8_Zr_0.1_Mn_0.1_O_3_	Co-precipitation	15.0	[75]
La(Mn,Pd)O_3_ (2.9 wt.% Pd)	Citrate method	12.0	[67]
La(Mn,Pd)O_3_ (2.32 wt.% Pd)	Flame pyrolysis	32.0	[67]
La(Mn,Pd)O_3_ (2.37 wt.% Pd)	Solution combustion	1.0	[67]
La(Mn,Pd)O_3_ (2.11 wt.% Pd)	Ultrasonic spray combustion	39.0	[67]
La_0.9_Ce_0.1_MnO_3_	Citrate	32.0	[71]
La_0.9_Ce_0.1_MnO_3_	Flame-pyrolysis	84.0	[61]
(La-Ce)MnO_3_	Mechanochemical	19.0	[51]
PrMnO_3+x_	Co-precipitation	2.5	[69]
NdMnO_3+x_	Co-precipitation	2.4	[69]
NdMnO_3_	Citrate	20.0	[72]
SmMnO_3_		19.0	
Sm_0.9_Sr_0.1_MnO_3_		20.0	
Sm_0.7_Sr_0.3_MnO_3_		14.0	
Sm_0.5_Sr_0.5_MnO_3_		13.0	
La_0.9_Eu_0.1_MnO_3_	Citrate	26.4	[71]
GdMnO_3+x_	Co-precipitation	5.3	[69]
(Dy-Y)MnO_3_	Mechanochemical	14.0	[51]

^a^ 1D non-porous.

**Table 4:** The influence of the synthesis method on the specific surface area of lanthanide ferrite-based perovskite powders.

Catalyst	Preparation Method	SSA (m^2^/g)	Ref.
LaFeO_3_	Co-precipitation	10.0	[57]
LaFeO_3_	Co-precipitation	5.5	[3]
LaFeO_3+x_	Co-precipitation	3.5	[69]
LaFeO_3_	Co-precipitation	20.9	[76]
LaFeO_3_	Solid state reaction	3.1	[45]
LaFeO_3_	Citrate	2.9	[77]
LaFeO_3_	Citrate	3.0	[78]
LaFeO_3_	Citrate	19.5	[76]
LaFeO_3_	Citrate	21.0	[79]
LaFeO_3_	Combustion	3.0	[63]
LaFeO_3+x_	Flame-hydrolysis	15.6	[65]
LaFeO_3_	Flame-pyrolysis	38.0	[61]
LaFeO_3_	Ultrasound spray combustion	9.8	[64]
LaFe_0.9_Mg_0.1_O_3_	Citrate	4.3	[77]
LaFe_0.8_Mg_0.2_O_3_		5.5	
LaFe_0.7_Mg_0.3_O_3_		7.9	
LaFe_0.6_Mg_0.4_O_3_		9.7	
LaFe_0.5_Mg_0.5_O_3_		5.3	
La_0.9_Ca_0.1_FeO_3_	Co-precipitation	14.0	[76]
La_0.9_Ca_0.1_FeO_3_	Citrate	6.0	[78]
La_0.9_Ca_0.1_FeO_3_	Citrate	17.8	[76]
La_0.9_Ca_0.1_FeO_3_	Citrate	38.0	[79]
La_0.8_Ca_0.2_FeO_3_	Co-precipitation	8.3	[76]
La_0.8_Ca_0.2_FeO_3_	Citrate	5.0	[78]
La_0.8_Ca_0.2_FeO_3_	Citrate	38.6	[76]
La_0.8_Ca_0.2_FeO_3_	Citrate	38.0	[79]
La_0.7_Ca_0.3_FeO_3_	Co-precipitation	5.4	[76]
La_0.7_Ca_0.3_FeO_3_	Citrate	3.0	[78]
La_0.7_Ca_0.3_FeO_3_	Citrate	38.6	[76]
La_0.7_Ca_0.3_FeO_3_	Citrate	38.0	[79]
La_0.6_Ca_0.4_FeO_3_	Co-precipitation	8.3	[76]
La_0.6_Ca_0.4_FeO_3_	Citrate	31.2	[76]
La_0.6_Ca_0.4_FeO_3_	Citrate	5.0	[78]
La_0.6_Ca_0.4_FeO_3_	Citrate	33.0	[79]
La_0.5_Ca_0.5_FeO_3_	Citrate	0.7	[78]
La_0.8_Sr_0.2_FeO_3_	Solid state reaction	4.7	[45]
La(Fe,Pd)O_3_ (2.4 wt.% Pd)	Citrate	14.0	[67]
La(Fe,Pd)O_3_ (2.28 wt.% Pd)	Flame pyrolysis	22.0	
La(Fe,Pd)O_3_ (1.25 wt.% Pd)	Ultrasonic spray combustion	27.0	
La(Fe,Pd)O_3_ (2.47 wt.% Pd)	Solution combustion	1.6	
(La-Ce)FeO_3_	Mechanochemical	5.3	[51]
PrFeO_3+x_	Co-precipitation	5.8	[69]
NdFeO_3+x_	Co-precipitation	4.5	[69]
NdFeO_3_	Citrate	2.3	[77]
SmFeO_3_	Citrate	4.3	[77]
GdFeO_3+x_	Co-precipitation	5.6	[69]
(Dy-Y)FeO_3_	Mechanochemical	8.6	[51]

**Table 5:** The effect of the preparation methods on the SSA of supported perovskites.

Catalyst	Support	Morphology of the Support	Preparation Method	SSA (m^2^/g)	Ref.
LaMnO_3_	foil Fe_2_Cr_20_Al_5_	monolith	Wet impregnation	23.3	[80]
LaMnO_3_	ZrO_2_	powder	Solution combustion method	132.5	[82]
30% LaMnO_3_	(5%La_2_O_3_/Al_2_O_3_)	powder	Deposition precipitation	88.0	[70]
20% LaMnO_3_	MgO			25.0	[70]
La_0.8_Sr_0.2_MnO_3+x_	MgAl_2_O_4_	powder	Wet impregnation	34.0	[73]
La_0.8_Sr_0.2_MnO_3+x_	NiAl_2_O_4_			22.0	
La_0.8_Sr_0.2_MnO_3+x_	CoAl_2_O_4_			18.0	
La_0.95_Ag_0.05_MnO_3_	foil Fe_2_Cr_20_Al_5_	monolith	Wet impregnation	27.4	[80]
La_0.9_Ag_0.1_MnO_3_				30.9	
La_0.8_Ag_0.2_MnO_3_				29.4	
La_0.7_Ag_0.3_MnO_3_				31.5	
LaFeO_3_	FeCr(20%)Al(5%)	monolith	Citrate method	7.7	[81]
La_0.66_Sr_0.34_Ni_0.29_Co_0.69_Mn_0.02_O_3_(20%)	(47% Al_2_O_3_–52% SiO_2_)	fiber	Freeze-drying	18.0	[83]
La_0.66_Sr_0.34_Ni_0.29_Co_0.69_Mn_0.02_O_3_(15%)	(47% Al_2_O_3_–52% SiO_2_)			23.0	
La_0.66_Sr_0.34_Ni_0.29_Co_0.69_Fe_0.02_O_3_ (27%)	(95% Al_2_O_3_–5% SiO_2_)			27.0	
La_0.66_Sr_0.34_Ni_0.29_Co_0.69_Fe_0.02_O_3_ (14%)	(95% Al_2_O_3_–5% SiO_2_)			22.0	
La_0.66_Sr_0.34_Ni_0.29_Co_0.69_Fe_0.02_O_3_(15%)	(47% Al_2_O_3_–52% SiO_2_)			24.0	
La_0.66_Sr_0.34_Ni_0.29_Co_0.69_Fe_0.02_O_3_(12%)	(47% Al_2_O_3_–52% SiO_2_)			27.0	

**Table 6:** Experimental conditions for different types of perovskite materials grown by PLD and PLD-RF techniques and the thickness of the films obtained.

Perovskite	Support	λ (nm)/ν (Hz)	Fluence (J/cm^2^)	P_O2_^a^ (mBar)	T ^b^ (°C)	Film Thickness (nm)	Ref.
BaTiO_3_	MgO (001)	248/10	2–4	4 × 10^−3^	1000	115	[88,89]
BaTiO_3_	SrTiO_3_ (001)	248/10	2–4	3 × 10^−1^	800	115	[88,89]
BaTiO_3_	SrTiO_3_ (001)	248/10	2–4	2 × 10^−3^	800	220	[88,89]
SrTiO_3_	LaAlO_3_ (100)	248/5	1.3	1.7 × 10^−4^	660	-	[90]
BaZrO_3_	Si	193/1–3	5.2	0.2	605	1–53	[91]
BaZrO_3_	SrTiO_3_ (100)	193/1–3	5.2	0.2	650	1–53	[91]
Y_1-x_Sr_x_MnO_3_ (x = 1, 0.9, 0.8, 0.7)	SrTiO_3_ (100)	248/3	2	0.01–0.13	800	0.6–0.65	[92]
Y_1-x_Sr_x_MnO_3_ (x = 1, 0.9, 0.8, 0.7)	LaAlO_3_ (100)	248/3	2	0.01–0.13	800	0.6–0.65	[92]
Y_1-x_Sr_x_MnO_3_ (x = 1, 0.9, 0.8, 0.7)	NdGaO_3_ (101)	248/3	2	0.01–0.13	800	0.6–0.65	[92]
La_0.67_Ca_0.33_MnO_3_	LaAlO_3_	-/5	n.s. ^d^	0.26	500–700	100	[93]
La_0.67_Ca_0.33_MnO_3_ *RF* ^c^	LaAlO_3_ (100)	n.s.	n.s.	Pressure of 0.053 (60:40 = Ar:O_2_ or pure Ar)	850	n.s.	[93]
La_0.67_Ca_0.33_MnO_3_ *RF* ^c^	NdGaO_3_ (110)	n.s.	n.s.	Presure of 0.053 (60:40 = Ar:O_2_ or pure Ar)	850	n.s.	[93]
La_0.67_Ca_0.33_MnO_3_	NdGaO_3_	248/8	0.2–0.25	0.13	600–800	85	[94]
La_0.67_Ca_0.33_MnO_3_ *RF* ^c^	SrTiO_3_ (100)	n.s.	n.s.	Presure of 0.053 (60:40 = Ar:O_2_ or pure Ar)	850	n.s.	[93]
SrFeO_3_	SrTiO_3_ (111)	248/2	2.3	0.13	700	n.s.	[95]
SrTi_1−x_Fe_x_O_3−y_ (x = 0.2–0.5)	Sapphire (Al_2_O_3_)	248/8	1.5	0.13	700	200–300	[96]
LaNiO_3_	SrTiO_3_ (100)	248/5	2	0.4	825	100	[97]
LaNiO_3_	LaAlO_3_ (100)	248/5	1.3	0.35	660	n.s.	[90]
Y(Ni_0.5_Mn_0.5_)O_3_	SrTiO_3_ (001)	248/2	1.5	0.6	550–850	70	[98]
Y(Ni_0.5_Mn_0.5_)O_3_	SrTiO_3_ (110)	248/2–20	2	0.6	550–850	70	[98]
Y(Ni_0.5_Mn_0.5_)O_3_	SrTiO_3_ (111)	248/2–20	2	0.6	550–850	70	[98]
Ca_0.25_Cu_0.75_TiO_3_	SrTiO_3_ (100)	248/10	3	0.16	600–800	250	[99]
SrRuO_3_	SrTiO_3_ (100)	193/1–3	5.2	0.2	590–650	n.s.	[91]
SrRuO_3_	SrTiO_3_ (111)	248	2.3	0.13	700	n.s.	[95]
PbTiO_3_	Si (100)	248/5	8	0.13–0.2	530	530	[100]
PbZr_0.2_Ti_0.8_ O_3_	SrRuO_3_/SrTiO_3_ (100)	193/1–3	5.2	0.2	587	n.s.	[91]
Pb(Zr_0.45_Ti_0.55_)O_3_	Pb(Zr_0.45_Ti_0.55_)O_3_/Pt/Ti/SiO_2_/Si prepared by citrate method	248/10	1.2	1.3 × 10^−4^	RT ^e^	600	[101]
BiFeO_3_	Pt/TiO_2_/SiO_2_/Si	355/2.5	n.s.	0.07	450	300	[102,103]
BiFeO_3_	SrTiO_3_ (001)	355/2.5	n.s.	0.01	580	70	[102,103]
BiFeO_3_	Pt(111)/TiO_2_/SiO_2_/Si(100)	248/5	2.5	n.s.	450	230	[104]
BiFeO_3_	SrTiO_3_ (100)	248/5	2	0.07	750	400	[105]
BiFeO_3_	SrRuO_3_/SrTiO_3_	248	n.s.	0.07	550–800	280–300	[106]
BiFeO_3_	Nb-doped SrTiO_3_ (100)	n.s.	n.s.	0.14	650	106.5	[107,108]
BiFeO_3_	SrTiO_3_ (100)	n.s.	n.s.	0.14	650	54.3	[107,108]
BiFeO_3_	DyScO_3_ (110)	n.s.	n.s.	0.14	650	34.1	[107,108]
BiFeO_3_	Nb-doped SrTiO_3_ (100)	248/5	n.s.	0.04–0.4	670	30–80	[109]
BiFeO_3_	Pt/TiO_2_/SiO_2_/Si	248/10	2.5	n.s.	RT ^e^	150	[110]
BiFeO_3_	Nb-doped SrTiO_3_ (100)	248	n.s.	0.02	500	10	[111]
BiFeO_3_	SrRuO_3_/SrTiO_3_ (001)	248	n.s.	0.01–0.26	650–750	n.s.	[112]
BiFeO_3_	Si (100)	248/5	n.s.	n.s.	670	50–100	[113]
BiFeO_3_	Pt/TiO_2_/SiO_2_/Si	248/3	1.5	0.53	625	100	[114,115]
BiFeO_3_	SrRuO_3_/SrTiO_3_ (111)	248/3	1.5	0.53	625	100	[114,115]
BiFeO_3_	Pt coated Al_2_O_3_	248/3	1.5	0.53	625	100	[114,115]
(1-x)BiFeO_3_-xPbTiO_3_	PbTiO_3_/Pt/Ti/SiO_2_/Si	248/5	2.5	0.2	550	230	[116]
(1-x)BiFeO_3_-xPbTiO_3_ (x = 0.7, 0.8, 0.9)	Pt/Ti/SiO_2_	248/5	6	0.2	545	400	[117]
LaCo_1-x_Cr_x_O_3_	LaAlO_3_ (100)	248/15	n.s.	0.083	650	450–530	[118]
LaMnO_3_	SrTiO_3_ (001)	n.s.	n.s.	0.013	700	30	[119]
LaMnO_3_	LaAlO_3_ (001)	248/2	n.s.	0.01	700	14–90	[120]
La_0.8_Ca_0.2_MnO_3_	LaAlO_3_ (100)	248/5	3.2	0.4	800	200	[121]
La_0.7_Sr_0.3_MnO_3_	Si (100)	248/10	n.s.	0.27	650	200	[122]
La_0.67_Sr_0.33_MnO_3_	LaAlO_3_	308/5	2	0.27	500–800	130	[123]
La_0.67_Sr_0.33_MnO_3_	SrTiO_3_	308/5	2	0.27	500–800	130	[123]
LaFeO_3_	SrTiO_3_ (100)	248/4	1.9	0.4	670	25–35	[124]
LaFeO_3_	GdScO_3_ (110)	248	1	0.13	700	100	[125]
LaFeO_3_	SrTiO_3_ (100)	248/4	2.4	0.2	670	54	[126]
LaFeO_3_	SrTiO_3_ (100)	248/5	5.5	0.07	800	400	[127]
LaFeO_3_	Nb-doped SrTiO_3_	193/5	2.2	0.05–0.9	750	n.s.	[128]
LaFeO_3_	SrTiO_3_ (100)	248/10	0.2	4 × 10^−5^	700	65	[129]
LaFeO_3_	LaAlO_3_ (100)	248/10	0.2	4 × 10^−5^	700	65	[129]
La_1-x_Sr_x_CoO_3_	Si (001)	266/10	2	0.05	740	120	[130]
La_1-x_Sr_x_CoO_3_	MgO (001)	266/10	2	0.05	740	120	[130]
La_1-x_Sr_x_CoO_3_ (x = 0, 0.1, 0.2)	Si (100)	248/10	2	0.13	600	n.s.	[131]
La_1-x_Sr_x_CoO_3_ (x = 0, 0.1, 0.2)	Si (100)	266/10	2	0.05	660	100	[132]
Sr_1-x_La_x_Ru_1-x_Fe_x_O_3_ (x = 0.05, 0.1, 0.2, 0.3)	SrTiO_3_ (100)	248/4	2.5	0.2–0.33	750	60	[133]
Bi_0.9_La_0.1_Fe_0.95_Mn_0.05_O_3_	Pt (111)/Ti/SiO2/Si	248	1.5	7 × 10^−3^	450–650	250	[134]
(Bi_1-x_La_x_)(Fe_1-x_Al_x_)O_3_ (x = 0, 0.1, 0.2, 0.3, 0.4)	Nb-doped SrTiO_3_ (001)	266	n.s.	0.065	600	n.s.	[135]
La_0.8_Ce_0.2_MnO_3_	LaAlO_3_ (001)	248/8	2	0.4	800	150	[136]
Bi_1-x_Pr_x_FeO_3_ (x = 0, 0.05, 0.1, 0.15)	Pt/SiO_2_	355/5	2.5	n.s.	450	200	[137]
NdNiO_3_	MgO (100)	248/10	1.5	0.15	675	500	[138]
NdNiO_3_	SrTiO_3_ (100)	248/10	1.5	0.15	675	500	[138]
NdNiO_3_	NdGaO_3_ (110)	248/10	1.5	0.15	675	500	[138]
NdNiO3	NdGaO_3_ (001)	-/10	1.9	3	900	30–50	[139]
Bi_0.9_Sm_0.1_Fe_0.95_Co_0.05_O_3_	Pt/TiO_2_/SiO_2_/Si	248/10	2–5	0.13	700–750	300–360	[140]
Bi_0.9_Sm_0.1_Fe_0.95_Co_0.05_O_3_	Pt/TiO_2_/SiO_2_/Si	248/10	2–5	0.13	700–750	300–360	[140]
Bi_1-x_Sm_x_FeO_3_ (x = 0.05, 0.1, 0.12, 0.14, 0.16)	Pt (111)/SiO_2_	355/5	n.s.	0.04	450	200	[141]
TmMnO_3_	SrTiO_3_ (110)	248	n.s.	0.1	940	20	[142]

^a^ P_O2_—the oxygen pressure during the deposition process; ^b^ T—the substrate temperature during the deposition process; ^c^ *RF*—prepared via PLD-RF; ^d^ n.s.—not specified; ^e^ RT—room temperature.

**Table 7:** Perovskites prepared by MAPLE.

Material	Target Concentration	Substrate	λ (nm)/ ν (Hz)	Fluence (J/cm^2^)	P_N2_ (mbar)	D_T-S_ (cm)	Ref.
rGO/BiFeO_3_	3 wt.% BiFeO_3_ 5 wt.% GO	F-doped SnO_2_	266/10	0.4	0.2	4	[148]
rGO/LaFeO_3_	3 wt.% LaFeO_3_ 5 wt.% GO	F-doped SnO_2_	266/10	0.4	0.2	4	[148]
CH_3_NH_3_PbI_3_	PbI_2_:MAI = 1:3	ITO	IR/2	0.125–0.135	1 × 10^−3^	7	[149]
CH_3_NH_3_PbI_3_	PbI_2_:MAI = 1:1 in DMSO and MEG	FTO/NiO_x_	-	-	-	-	[150]

Abbreviations: D_T-S_—the distance between the material target and the substrate; rGO—reduced graphene oxide; MAI—methylammonium iodide; ITO—indium tin oxide; IR—infrared radiation; DMSO—dimethyl sulfoxide; MEG—ethylene glycol; FTO—fluorine-doped tin oxide.

**Table 8:** Photoelectrodes based on perovskites for photoelectrochemical water-splitting reaction.

Material	Electrode Type	E_g_^a^ (eV)	Electrolyte/Light Source/Intensity (mW/cm^2^)	Performance	Stability	Ref.
BaTiO_3_	Photo-anode	3.11	0.1 M NaOH (pH = 13)/Xe arc UV-Vis lamp/180	0.5 V_SCE_: J_ph_ ≈ 0.07 mA/cm^2^;	n.s.^b^	[188]
PbTiO_3_-TiO_2_	Photo-anode	2.78–3.6	0.1 M KOH/Xe lamp Vis light	1.23 V_RHE_: J_ph_ ≈ 0.3 mA/cm^2^ IPCE^c^ ≈ 70% at 380 nm IPCE ≈ 38% at 420 nm IPCE < 1% at 500 nm Onset potential 0.3 V_RHE_	Stable after 300 s	[189]
SrTiO_3_ nanocubes	Photo-anode	3.43	0.1 Na_2_SO_4_ (pH = 7)/AM1.5/100	0 V_Ag/AgCl_: J_ph_ ≈ 0.5 uA/cm^2^ 0.9 V_Ag/AgCl_: J_ph_ ≈ 4 uA/cm^2^; 60 μmol/h O_2_	n.s.	[190]
SrTiO_3_	Photo-anode	3.4	0.1 M Na_2_SO_4/_AM1.5/100	0 V_Ag/AgCl_: J_ph_ ≈ 50 μA/cm^2^ 1.5 V_Ag/AgCl_: J_ph_ ≈ 0.5 mA/cm^2^ IPCE ≈ 10% at 350 nm IPCE < 1% for λ > 400 nm	n.s.	[191]
SrTiO_3_—carbon quantum dots	Photo-anode	n.s.	0.1 M Na_2_SO_4_/AM1.5/100	0 V_Ag/AgCl_: J_ph_ ≈ 110 μA/cm^2^ 1.5 V_Ag/AgCl_: J_ph_ ≈ 1.7 mA/cm^2^ IPCE ≈ 14% at 350 nm IPCE ≈ 1% at 860 nm	n.s.	[192]
BiFeO_3_	Photo-anode	2.1	0.2 M Na_2_SO_4/_sunlight 300 W Xenon lamp	0.8 V_Ag/AgCl_: J_ph_ ≈ 0.12 μA/cm^2^	Stable after 400 s	[201]
H_2_ treated BiFeO_3_	Photo-anode	2.0	0.2 M Na_2_SO_4/_sunlight, 300 W Xenon lamp	0.8 V_Ag/AgCl_: J_ph_ ≈ 0.69 μA/cm^2^	Stable after 400 s	[201]
BiFeO_3_	Photo-anode	2.4	0.2 M Na_2_SO_4_ (pH = 6.5)/AM1.5/100	1 V_Ag/AgCl_: J_ph_≈ 0.17 mA/cm^2^ IPCE≈ 17% at 420 nm Onset potential 0.1 V_Ag/AgCl_	n.s.	[202]
Ni-B/BiFeO_3_	Photo-anode	n.s.	0.1 M potassium borate (pH = 9.2)	1 V_Ag/AgCl_: J_ph_ ≈ 0.72 mA/cm^2^	Stable after 3 h	[202]
BFO/SrRuO_3_	Photo-anode	2.74	1M Na_2_SO_4_/250 mW/cm^2^	0.64 V_Ag/AgCl_: J_ph_ ≈ 10 μA/cm^2^ Onset potential 0.18 V_Ag/AgCl_	n.s.	[221]
Ti-doped BiFeO_3_	Photo-anode	1.97	1 M NaOH/300W UV Xe lamp	1.23 V_RHE_: J_ph_ ≈ 0.04mA/cm^2^ Onset potential 0.81 V_RHE_	Stable after 3600 s	[208]
Y-doped BiFeO_3_	Photo-anode	n.s.	0.5 M NaOH (pH = 13)/laser diode 405 nm (5 mW)	1.4 V_RHE_: J_ph_ = 0.72 mA/cm^2^	Stable after 900 s	[209]
BiFeO_3_/TiO_2_/FTO	Photo-anode	n.s.	1M NaOH/300 W Xenon lamp Vis light	1.5 V_SCE_: J_ph_ ≈ 15 mA/cm^2^ Onset potential ≈ 0.6 V_SCE_	Stable after 300 s	[222]
BiFeO_3_/TiO_2_/FTO	Photo-anode	n.s.	1M NaOH/AM1.5/100	1.5 V_SCE_: J_ph_ ≈ 17 mA/cm^2^ Onset potential ≈ 0.6 V_SCE_	Stable after 300 s	[222]
WO_3_/BiVO_4_/BiFeO_3_	Photo-anode	n.s.	0.5M Na_2_SO_4_/AM1.5/100	2.53 V_RHE_: J_ph_ = 46.9 mA/cm^2^	Stable after 200 s	[212]
LaFeO_3_	Photo-cathode	2.16	0.1 M NaOH (pH = 13)/AM1.5/100	0.73 V_RHE_: J_ph_ = −0.1 mA/cm^2^	Stable after 1 h	[223]
LaFeO_3_	Photo-cathode	2.16	0.1 M NaOH (pH = 13)/AM1.5/100	0.5 V_RHE_: J_ph_ ≈ −0.2 mA/cm^2^	Stable after 1 h	[223]
LaFeO_3_	Photo-anode	2.08	0.1 M KOH/500 W Xenon lamp, Vis light/100	1.1 V_Ag/AgCl_: J_ph_≈ 1.2 mA/cm^2^ Onset potential 0.48 V_Ag/AgCl_	Stable after 330 s	[220]
LaFeO_3_	Photo-cathode	2.4	0.1 M NaOH	0.26 V_RHE_: J_ph_ ≈ 0.16 mA/cm^2^ Onset potential 1.2 V_RHE_	Stable after 21 h	[224]
LaFeO_3_	Photo-cathode	1.95	1M Na_2_SO_4_/AM1.5/100	1.7 V_Ag/AgCl_: J_ph_ ≈ 8.2 mA/cm^2^	decreases to 50% after 30 min	[225]
LaFeO_3_	Photo-anode	2.07	0.1 M NaOH (pH = 13)/laser diode 405 nm (5mW)	1 V_Ag/AgCl_: J_ph_ = 1.6 mA/cm^2^	n.s.	[128]
LaFe_0.9_Mn_0.1_O_3_	Photo-anode	~2.08	0.1 M KOH/500 W Xenon lamp, Vis light/100	1.1V_Ag/AgCl_: J_ph_ ≈ 1.5 mA/cm^2^ Onset potential 0.34 V	Stable after 330 s	[220]
LaFe_0.9_Co_0.1_O_3_	Photo-anode	~2.08	0.1 M KOH/500 W Xenon lamp, Vis light/100	1.1 V_Ag/AgCl_: J_ph_ ≈ 1.8 mA/cm^2^ Onset potential 0.27 V	Stable after 330 s	[220]
LaFe_0.9_Cu_0.1_O_3_	Photo-anode	~2.08	0.1 M KOH/500 W Xenon lamp, Vis light/100	1.1 V_Ag/AgCl_: J_ph_ ≈ 2 mA/cm^2^ Onset potential 0.27 V	Stable after 330 s	[220]
p-LaFeO_3_/n-Fe_2_O_3_	Photo-cathode/ Photo-anode	n.s.	1M NaOH/AM1.5/100	0 V_RHE:_ J_ph_ = 64.5 μA/cm^2^ 11.5 μmol/h H_2_ 5.7 μmol/h O_2_	Stable after 120 h	[218]
LaFeO_3_	Photo-cathode	2.4	0.1 M NaOH/AM1.5/100	0.6 V_RHE_: J_ph_ ≈ −0.04 mA/cm^2^	n.s.	[226]
Ag-LaFeO_3_	Photo-cathode	n.s.	1 M NaOH/AM1.5/100	0.6 V_RHE_: J_ph_ ≈ −0.074 mA/cm^2^	n.s.	[226]
LaFeO_3_	Photo-cathode	2.07	0.1 M Na_2_SO_4_/AM1.5/100	0.6 V_RHE_: J_ph_ ≈ −4.78 μA/cm^2^	decreases to 88.6% after 2750 s	[227]
FTO/Au/LaFeO_3_	Photo-cathode	n.s.	0.1 M Na_2_SO_4_/AM1.5/100	0.6 V_RHE_: J_ph_ ≈ −19.60 μA/cm^2^	decreases to 91% after 2750 s	[227]

^a^ E_g_—band gap; ^b^ n.s.—not specified; ^c^ ICPE—Incident Photon to Current Efficiency.

**Table 9:** Methane combustion activity of undoped perovskite-type materials expressed as the temperature where 50% conversion is reached (T_50_).

Catalyst	SSA (m^2^/g)	Reaction Conditions	T_50_ (°C)	E_a_^a^ (kJ/mol)	Ref.
LaCoO_3_	3.0	2 vol. % CH_4_ in air, 45,000–50,000/h	525	22.1	[45]
LaCoO_3_	3.5	1 vol. % CH_4_, 4 vol. % O_2_ in He, 135,000/h	709	~104	[56]
LaCoO_3_	5.7	3 vol. % CH_4_, 7.2 vol. % O_2_ in N_2_, 113 cm^3^/min, 0.5 g catalyst	~647	n.s. ^b^	[63]
LaCoO_3_	8	1 vol. % CH_4_ in air	545	n.s.	[57]
LaCoO_3_	15.0	0.4 vol. % CH_4_, 10 vol. % O_2_ in N_2_, 40,000 Ncm^3^/h x g_cat_	~567	n.s.	[50]
LaCoO_3+x_	15.6–22.8	10 cm^3^ (1.04 vol. % CH_4_ in He) with 10 cm^3^ of air, 0.2 g catalyst	466	n.s.	[65]
LaCoO_3_	11.3	1 vol. % CH_4_ in air, 45,000 mL/(h g_cat_), 0.1 g catalyst	600	97	[58]
LaCoO_3_	6.0	1 vol. % CH_4_, 4 vol.% O_2_ in He, 20,000–200,000/h, 0.1 g catalyst	~600	n.s.	[59]
LaCoO_3_	43	0.34 vol.% CH_4_, 33.3 vol.% air in He, 30 Ncm^3^/min, 0.15 g catalyst	449	n.s.	[61]
LaCoO_3_	5.5	1 vol.% CH_4_, 4 vol.% O_2_ in He, 14,150/h, 0.1 g catalyst	560	n.s.	[64]
PrCoO_3_	5.1	1 vol.% CH4, 4 vol.% O_2_ in He, 135,000/h	903	~110	[56]
NdCoO_3_	1.6		658	~103	
GdCoO_3_	2.1		733	~99	
LaMnO_3_	4.0	2 vol.% CH_4_ in air, 45,000–5000/h	579	21.8	[45]
LaMnO_3_	~15	1.5 vol.% CH_4_, 4.2 vol.% O_2_ in He), 200 cm^3^/min, 0.004 g catalyst	n.s.	73	[3]
LaMnO_3_	5.6	3.2 vol.% CH_4_, 12.8 vol.% O_2_ in Ar, 73.5 mL/min	457	92	[49]
LaMnO_3+x_	8.0	1 vol.% CH_4_, 4 vol.% O_2_ in He, 135,000/h	682	~82	[69]
LaMnO_3_	7	1 vol.% CH_4_ in air	580	n.s.	[57]
LaMnO_3_	n.s.	1 vol.% CH_4_ in air, ~50,000 cm^3^/(h g_cat_)	~577	n.s.	[75]
LaMnO_3_	20.0	0.5 vol.% CH_4_, 10 vol.% air in N_2_, 40 Ncm^3^/min, 0.2 g catalyst	~440	n.s.	[71]
LaMnO_3_	22.0	0.4 vol.% CH_4_, 10 vol.% O_2_ in N_2_, 40,000 Ncm^3^/(h g_cat_)	~506	n.s.	[50]
LaMnO_3_	20.0	0.4 vol.% CH_4_, 10 vol.% O_2_ in N_2_	~507	23.3	[72]
LaMnO_3_	11.0	0.4 vol.% CH_4_, 10 vol.% O_2_ in N_2_	575	24.4	[70]
LaMnO_3+x_	15.6–22.8	10 cm^3^ (1.04 vol.% CH_4_ in He) with 10 cm^3^ of air, 0.2g catalyst	489	n.s.	[65]
LaMnO_3_	n.s.	0.4 vol.% CH_4_, 10 vol.% O_2_ in N_2_, 40,000 Ncm^3^/(h g_cat_), 0.4 g catalyst	511	97.5	[238]
LaMnO_3_	22.0	0.4 vol.% CH_4_, 10 vol.% O_2_ in N_2_, 40,000 cm^3^/(h g_cat_), 0.4 g catalyst	~507	23.3	[55]
LaMnO_3_	56	0.34 vol.% CH_4_, 33.3 vol.% air in He, 30 Ncm^3^/min, 0.15 g catalyst	435	n.s.	[61]
LaMnO_3_	21.8	1 vol.% CH_4_, 4 vol.% O_2_ in He, 14,150/h, 0.1 g catalyst	515	n.s.	[64]
LaMnO_3_	68	1 vol.% CH_4,_ 4 vol.% O_2_ in N_2_, 40,000 cm^3^/(h g_cat_), 0.15 g catalyst	446	n.s.	[66]
30% LaMnO_3/_ (5% La_2_O_3_/Al_2_O_3_)	88.0	0.4 vol.% CH_4_, 10 vol.% O_2_ in N_2_	532	18.2	[70]
20% LaMnO_3_/MgO	25.0		533	23.3	
LaMnO_3_-ZrO_2_	132.5	2 vol.% CH_4_, 16 vol.% O_2_ in He, 50 Ncm^3^/min, 0.1 g catalyst	595	n.s.	[82]
Pd/LaMnO_3_-ZrO_2_	74.6		570	n.s.	
0.5% Pt/LaMnO_3_	63	0.34 vol.% CH_4_, 33.3 vol.% air in He, 30 Ncm^3^/min, 0.15 g catalyst	426	n.s.	[61]
0.5% Pd/LaMnO_3_	53		445	n.s.	
LaMnO_3_/foil Fe_2_Cr_20_Al_5_	23.3	1 vol.% CH_4_ in air, 64,410 cm^3^/(h g_cat_), 25.7 g catalyst	566	n.s.	[80]
PrMnO_3+x_	2.5	1 vol.% CH_4_, 4 vol.% O_2_ in He, 135,000/h	711	~89	[69]
NdMnO_3+x_	2.4		695	~83	
NdMnO_3_	20.0	0.4 vol.% CH_4_, 10 vol.% O_2_ in N_2_	~587	19.3	[72]
GdMnO_3+x_	5.3	1 vol.% CH_4_, 4 vol.% O_2_ in He, 135,000/h	677	~79	[69]
SmMnO_3_	19.0	0.4 vol.% CH_4_, 10 vol.% O_2_ in N_2_	~527	17.1	[72]
LaFeO_3_	3.1	2 vol.% CH_4_ in air, 45,000–5000/h	571	18.2	[45]
LaFeO_3_	5.5	1.5 vol.% CH_4_, 4.2 vol.% O_2_ in He, 200 cm^3^/min, 0.004g catalyst	n.s.	75	[3]
LaFeO_3_	3.0	n.s.	~672	n.s.	[63]
LaFeO_3_	10	1 vol.% CH_4_ in air	545	n.s.	[57]
LaFeO_3_	2.9	0.4 vol.% CH_4_, 10 vol.% O_2_ in N_2_, 40,000 Ncm^3^/(h g_cat_)	529	20.76	[77]
LaFeO_3_	3.0	0.4 vol.% CH_4_, 10 vol.% O_2_ in N_2_, 40,000 Ncm^3^/(h g_cat_), 0.4 g catalyst	529	21.07	[78]
LaFeO_3_	20.9	n.s.	608	n.s.	[76]
LaFeO_3_	19.5		508		
LaFeO_3_	38	0.34 vol.% CH_4_, 33.3 vol.% air in He, 30 Ncm^3^/min, 0.15 g catalyst	495	n.s.	[61]
LaFeO_3_	9.8	1 vol.% CH_4_, 4 vol.% O_2_ in He, 14,150/h, 0.1 g catalyst	625	n.s.	[64]
LaFeO_3_	21.0	37,000 ppmv CH_4_, 23.22 vol.% O_2_ in He	512	105.7	[79]
LaFeO_3+x_	15.6	10 cm^3^ (1.04 vol.% CH_4_ in He) with 10 cm^3^ of air, 0.2g catalyst	472	n.s.	[65]
LaFeO_3+x_	3.5	1 vol.% CH_4_, 4 vol.% O_2_ in He, 135,000/h	678	~105	[69]
LaFeO_3_/FeCr(20%)Al(5%)	7.7	1 vol.% CH_4_ in air, 5800/h	577	101.8	[81]
PrFeO_3+x_	5.8	1 vol.% CH_4_, 4 vol.% O_2_ in He, 135,000/h	717	86	[69]
NdFeO_3+x_	4.5		718	~109	
NdFeO_3_	2.3	0.4 vol.% CH_4_, 10 vol.% O_2_ in N_2_, 40,000 Ncm^3^/(h g_cat_)	556	20.76	[77]
GdFeO_3+x_	5.6	1 vol.% CH_4_, 4 vol.% O_2_ in He, 135,000/h	707	~89	[69]
SmFeO_3_	4.3	0.4 vol.% CH_4_, 10 vol.% O_2_ in N_2_, 40,000 Ncm^3^/(h g_cat_)	558	23.66	[77]
LaCuO_3_	0.6	2 vol.% CH_4_ in air, 45,000–5000/h	672	23.8	[45]
LaNiO_3_	4.8		702	19.4	
LaNiO_3_	n.s.	1.5 vol.% CH_4_, 4.2 vol.% O_2_ in He, 200 cm^3^/min, 0.004 g catalyst	n.s.	79	[3]
LaNiO_3_	17.0	0.4 vol.% CH_4_, 2 vol.% O_2_ in He, 60,000/h, 1.5 g catalyst	~600	18.7	[51]
LaCrO_3_	1.9	2 vol.% CH_4_ in air, 45,000–5000/h	780	28.8	[45]
LaCrO_3_	n.s.	1.5 vol.% CH_4_, 4.2 vol.% O_2_ in He, 200 cm^3^/min, 0.004g catalyst	n.s.	142	[3]
LaCrO_3_	~5–7	1.5 vol.%, 18 vol.% in He, 1.2cm^3^/s	692	n.s.	[47]
LaRuO_3_	n.s.	1.5 vol.% CH_4_, 4.2 vol.% O_2_ in He, 200 cm^3^/min, 0.004g catalyst	-	95	[3]
LaAlO_3_	4.0	0.4 vol.% CH_4_, 10 vol.% O_2_ in N_2_, 40,000 cm^3^/(h g_cat_), 0.4 g catalyst	~652	28.1	[55]
BaCeO_3_-ZrO_2_	45.6	2 vol.% CH_4_, 16 vol.% O_2_ in He, 50 Ncm^3^/min, 0.1 g catalyst	490	n.s.	[82]
Pd/BaCeO_3_-ZrO_2_	26.4		512	n.s.	
BaTiO_3_	0.4	5 vol.% CH_4_ in air, 16,000/h	744	85.8	[44]
PbTiO_3_	0.5		697	104.9	

^a^ Ea = activation energy; ^b^ n.s.—not specified.

**Table 10:** Performances of A-site-doped perovskite-type materials for the catalytic combustion of methane.

Catalyst	SSA (m^2^/g)	Reaction Conditions	T_50_ (°C)	E_a_ (kJ/mol)	Ref.
La_0.9_Sr_0.1_CoO_3_	52	0.34 vol.% CH_4_, 33.3 vol.% air in He, 30 Ncm^3^/min, 0.15 g catalyst	454	n.s. ^b^	[61]
La_0.8_Sr_0.2_CoO_3_	4.7	2 vol.% CH_4_ in air, 45,000–5000/h	518	21.3	[45]
La_0.8_Sr_0.2_CoO_3_	16.5	4 vol.% CH_4_ in air, 4.2–5 cm^3^/s, 1g catalyst	640 ^a^	n.s.	[53]
La_0.75_Sr_0.25_CoO_3_	n.s.	1.5 vol.% CH_4_, 4.2 vol.% O_2_ in He, 200 cm^3^/min, 0.004 g catalyst	n.s.	81	[3]
La_0.66_Sr_0.34_CoO_3_	17.4	4 vol% CH_4_ in air, 4.2–5 cm^3^/s, 1 g catalyst	675 ^a^	n.s.	[53]
La_0.6_Sr_0.4_CoO_3_	3.6	2 vol.% CH_4_ in air, 45,000–5000/h	570	19.0	[45]
La_0.5_Sr_0.5_CoO_3_	n.s.	1.5 vol.% CH_4_, 4.2 vol.% O_2_ in He), 200 cm^3^/min, 0.004g catalyst	n.s.	70	[3]
La_0.8_Ba_0.2_CoO_3_	5.1	2 vol.% CH_4_ in air, 45,000–5000/h	535	16.9	[45]
La_0.8_Ca_0.2_CoO_3_	2.0		606	18.1	
La_0.95_Ce_0.05_CoO_3_	8.7	1 vol.% CH_4_ in air, 45,000 mL/(h g_cat_), 0.1 g catalyst	532	86	[58]
La_0.9_Ce_0.1_CoO_3_	10.0		515	83	
La_0.9_Ce_0.1_CoO_3_	62	0.34 vol.% CH_4_, 33.3 vol.% air in He, 30 Ncm^3^/min, 0.15 g catalyst	447	n.s.	[61]
La_0.8_Ce_0.2_CoO_3_	3.1	2 vol.% CH_4_ in air, 45,000–5000/h	499	19.7	[45]
La_0.8_Ce_0.2_CoO_3_	14.2	1 vol.% CH_4_ in air, 45,000 mL/(h g_cat_), 0.1 g catalyst	520	97	[58]
La_0.7_Ce_0.3_CoO_3_	14.3		505	81	
La_0.6_Ce_0.4_CoO_3_	11.6		530	n.s.	
La_0.9_Sr_0.1_MnO_3_	51	0.34 vol.% CH_4_, 33.3 vol.% air in He, 30 Ncm^3^/min, 0.15 g catalyst	419	n.s.	[61]
La_0.8_Sr_0.2_MnO_3_	8.6	2 vol.% CH_4_ in air, 45,000–5000/h	510	19.7	[45]
La_0.8_Sr_0.2_MnO_3_	70	0.34 vol.% CH_4_, 33.3 vol.% air in He, 30 Ncm^3^/min, 0.15 g catalyst	434	n.s.	[61]
La_0.8_Sr_0.2_MnO_3+x_	5.0	1 vol.% CH_4_, 4 vol.% O_2_ in He, 135,000/h	624	104	[73]
La_0.8_Sr_0.2_MnO_3+x_/MgAl_2_O_4_	34.0		619	92	
La_0.8_Sr_0.2_MnO_3+x_/NiAl_2_O_4_	22.0		642	98	
La_0.8_Sr_0.2_MnO_3+x_/CoAl_2_O_4_	18.0		707	114	
La_0.75_Sr_0.25_MnO_3_	n.s.	1.5 vol.% CH_4_, 4.2 vol.% O_2_ in He), 200 cm^3^/min, 0.004 g catalyst	n.s.	65	[3]
La_0.6_Sr_0.4_MnO_3_	3.3	2 vol.% CH_4_ in air, 45,000–5000/h	482	20.1	[45]
La_0.6_Sr_0.4_MnO_3_	18.7	0.5 vol.% CH_4_, 10 vol.% air in N_2_, 40 Ncm^3^/min, 0.2 g catalyst	~470	n.s.	[71]
La_0.6_Sr_0.4_MnO_3_	4.32	5 vol.% CH_4_, 30 vol.% O_2_ in N_2_, 50,000 cm^3^/(h g_cat_), 0.05 g catalyst	480	136	[74]
La_0.6_Sr_0.4_MnO_3_	33.5		385	102	
La_0.6_Sr_0.4_MnO_3_	48.9		360	67.3	
La_0.5_Sr_0.5_MnO_3_	n.s.	1.5 vol.% CH_4_, 4.2 vol.% O_2_ in He), 200 cm^3^/min, 0.004 g catalyst	n.s.	60	[3]
La_0.8_Ca_0.2_MnO_3_	6.7	2 vol.% CH_4_ in air, 45,000–5000/h	543	18.9	[45]
La_0.9_Ce_0.1_MnO_3_	32	0.5 vol.% CH_4_, 10 vol.% air in N_2_, 40 Ncm^3^/min, 0.2 g catalyst	~440	n.s.	[71]
La_0.9_Ce_0.1_MnO_3_	84	0.34 vol.% CH_4_, 33.3 vol.% air in He, 30 Ncm^3^/min, 0.15 g catalyst	433	n.s.	[61]
(La-Ce)MnO_3_	19.0	0.4 vol.% CH_4_, 2 vol.% O_2_ in He, 60,000/h, 1.5g catalyst	~730	21	[51]
La_0.9_Eu_0.1_MnO_3_	26.4	0.5 vol.% CH_4_, 10 vol.% air in N_2_, 40 Ncm^3^/min, 0.2 g catalyst	~425	n.s.	[71]
Sm_0.9_Sr_0.1_MnO_3_	20.0	0.4 vol.% CH_4_, 10 vol.% O_2_ in N_2_	~557	20.8	[72]
Sm_0.7_Sr_0.3_MnO_3_	14.0		~527	18.6	
(Dy-Y)MnO_3_	14.0	0.4 vol.% CH_4_, 2 vol.% O_2_ in He, 60,000/h, 1.5g catalyst	~650	25.2	[51]
La_0.95_Ag_0.05_MnO_3_/ foil Fe_2_Cr_20_Al_5_	27.4	1 vol.% CH_4_ in air, 64,410 cm^3^/(h g_cat_), 25.7 g catalyst	n.s.	n.s.	[80]
La_0.9_Ag_0.1_MnO_3_/ foil Fe_2_Cr_20_Al_5_	30.9		n.s.	n.s.	
La_0.8_Ag_0.2_MnO_3_/ foil Fe_2_Cr_20_Al_5_	29.4		520	n.s.	
La_0.7_Ag_0.3_MnO_3_/ foil Fe_2_Cr_20_Al_5_	31.5		528	n.s.	
La_0.8_Sr_0.2_FeO_3_	4.7	2 vol.% CH_4_ in air, 45,000–5000/h	542	17.8	[45]
(La-Ce)FeO_3_	5.3	0.4 vol.% CH_4_, 2 vol.% O_2_ in He, 60,000/h, 1.5g catalyst	~700	26.0	[51]
La_0.9_Ca_0.1_FeO_3_	6.0	0.4 vol.% CH_4_, 10 vol.% O_2_ in N_2_, 40,000 Ncm^3^/(h g_cat_), 0.4 g catalyst	543	22.6	[78]
La_0.9_Ca_0.1_FeO_3_	14.0	n.s.	517	n.s.	[76]
La_0.9_Ca_0.1_FeO_3_	17.8		505	n.s.	
La_0.9_Ca_0.1_FeO_3_	38.0	37,000 ppmv CH_4_, 23.22 vol.% O_2_ in He	508	97.9	[79]
La_0.8_Ca_0.2_FeO_3_	5.0	0.4 vol.% CH_4_, 10 vol.% O_2_ in N_2_, 40,000 Ncm^3^/(h g_cat_), 0.4 g catalyst	537	22.6	[78]
La_0.8_Ca_0.2_FeO_3_	8.3	n.s.	530	n.s.	[76]
La_0.8_Ca_0.2_FeO_3_	38.6		503	n.s.	
La_0.8_Ca_0.2_FeO_3_	38.0	37,000 ppmv CH_4_, 23.22 vol.% O_2_ in He	502	95.5	[79]
La_0.7_Ca_0.3_FeO_3_	3.0	0.4 vol.% CH_4_, 10 vol.% O_2_ in N_2_, 40,000 Ncm^3^/(h g_cat_), 0.4 g catalyst	525	22.6	[78]
La_0.7_Ca_0.3_FeO_3_	5.4	n.s.	508	n.s.	[76]
La_0.7_Ca_0.3_FeO_3_	38.6		505	n.s.	
La_0.7_Ca_0.3_FeO_3_	38.0	37,000 ppmv CH_4_, 23.22 vol.% O_2_ in He	494	94.9	[79]
La_0.6_Ca_0.4_FeO_3_	5.0	0.4 vol.% CH_4_, 10 vol.% O_2_ in N_2_, 40,000 Ncm^3^/(h g_cat_), 0.4 g catalyst	541	22.6	[78]
La_0.6_Ca_0.4_FeO_3_	8.3	n.s.	511	n.s.	[76]
La_0.6_Ca_0.4_FeO_3_	31.2		511	n.s.	
La_0.6_Ca_0.4_FeO_3_	33.0	37,000 ppmv CH_4_, 23.22 vol.% O_2_ in He	487	94.9	[79]
La_0.5_Ca_0.5_FeO_3_	0.7	0.4 vol.% CH_4_, 10 vol.% O_2_ in N_2_, 40,000 Ncm^3^/(h g_cat_), 0.4 g catalyst	636	21.4	[78]
(Dy-Y)FeO_3_	8.6	0.4 vol.% CH_4_, 2 vol.% O_2_ in He, 60,000/h, 1.5 g catalyst	~750	31.3	[51]
(Dy-Y)NiO_3_	9.5		~670	28.4	

^a^ T_100_—the temperature corresponding to 100% methane conversion; ^b^ n.s.—not specified.

**Table 11:** Performances of B-site doped perovskite-type materials for the catalytic combustion of methane.

Catalyst	SSA (m^2^/g)	Reaction Conditions	T_50_	E_a_ (kJ/mol)	Ref.
LaCr_0.9_Mg_0.1_O_3_	~5–7	1.5 vol.%, 18 vol.% in He, 1.2 cm^3^/s	641	n.s. ^a^	[47]
LaCr_0.8_Mg_0.2_O_3_			647	n.s.	
LaCr_0.7_Mg_0.3_O_3_			594	n.s.	
LaCr_0.6_Mg_0.4_O_3_			562	n.s.	
LaCr_0.5_Mg_0.5_O_3_			553	n.s.	
LaCr_0.5_Mg_0.5_O_3_	6.08	2 vol.% CH_4_, 18 vol.% O_2_ in He, 50 Ncm^3^/min, 0.5 g catalyst	577	n.s.	[48]
LaCr_0.5_Mg_0.5_O_3_∙2MgO	13.6		552	n.s.	
LaCr_0.5_Mg_0.5_O_3_∙6MgO	24.2		545	n.s.	
LaCr_0.5_Mg_0.5_O_3_∙17MgO	36.9		529	n.s.	
LaAl_0.95_Mn_0.05_O_3_	8.0	0.4 vol.% CH_4_, 10 vol.% O_2_ in N_2_, 40,000 cm^3^/(h g_cat_), 0.4 g catalyst	~607	28.2	[55]
LaAl_0.9_Mn_0.1_O_3_	7.0		n.s.	n.s.	
LaAl_0.8_Mn_0.2_O_3_	25.0		~542	26.1	
LaAl_0.6_Mn_0.4_O_3_	25.0		~507	25.0	
LaAl_0.4_Mn_0.6_O_3_	26.0		~500	24.4	
LaAl_0.2_Mn_0.8_O_3_	33.0		~442	22.8	
LaMn_0.8_Cu_0.2_O_3_	19.0	0.4 vol.% CH_4_, 10 vol.% O_2_ in N_2_, 40,000 Ncm^3^/(h g_cat_)	~832	n.s.	[50]
LaMn_0.6_Cu_0.4_O_3_	14.0		~827	n.s.	
La(MnPd)O_3_ (2.32 wt.% Pd)	32	1 vol.% CH_4_, 4 vol.% O_2_ in He, 60,000/h	542	n.s.	[67]
La(MnPd)O_3_ (2.11 wt.% Pd)	39		482	n.s.	
LaFe_0.9_Mg_0.1_O_3_	4.3	0.4 vol.% CH_4_, 10 vol.% O_2_ in N_2_, 40,000 Ncm^3^/(h g_cat_)	547	23.39	[77]
LaFe_0.8_Mg_0.2_O_3_	5.5		541	23.39	
LaFe_0.7_Mg_0.3_O_3_	7.9		552	23.39	
LaFe_0.6_Mg_0.4_O_3_	9.7		565	25.55	
LaFe_0.5_Mg_0.5_O_3_	5.3		579	25.55	
LaFe_0.84_Cu_0.16_O_3_	4.0	3.2 vol.% CH_4_, 12.8 vol.% O_2_ in Ar, 73.5 mL/min	512	87	[49]
La(Fe,Pd)O_3_ (2.28 wt.% Pd)	22	1 vol.% CH_4_, 4 vol.% O_2_ in He, 60,000/h	545	n.s.	[67]
La(Fe,Pd)O_3_ (1.25 wt.% Pd)	27		584	n.s.	
La(Fe,Pd)O_3_ (2.47 wt.% Pd)	1.6		584	n.s.	
La(Fe,Pd)O_3_ (2.4 wt.% Pd)	14		565	n.s.	
LaCo_0.8_Cu_0.2_O_3_	21.0	0.4 vol.% CH_4_, 10 vol.% O_2_ in N_2_, 40,000 Ncm^3^/(h g_cat_)	~607	n.s.	[50]
LaCo_0.6_Cu_0.4_O_3_	13.0		~630	n.s.	
SrTi_0.8_Zr_0.1_Mn_0.1_O_3_	15	1 vol.% CH_4_ in air, ~50,000 cm^3^/(h g_cat_)	~587	n.s.	[75]

^a^ n.s.—not specified.

**Table 12:** Chemical structures of some organic pollutants.

No.	Compound	Abbreviation	Ref.
1	Rhodamine B C_28_H_31_ClN_2_O_3_ 479.01 g mol^−1^ 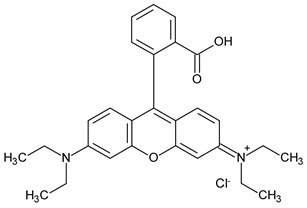	Rh B	[290]
2	Methyl orange C_14_H_14_N_3_NaO_3_S 327.33 g mol^−1^ 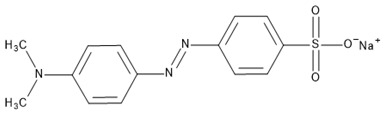	MO	[291]
3	Methylene blue C_16_H_18_ClN_3_S 319.85 g mol^−1^ 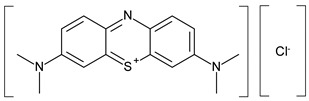	MB	[282]
4	Congo red C_32_H_22_N_6_Na_2_O_6_S_2_ 696.665 g mol^−1^ 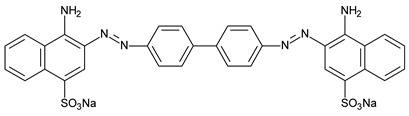	CR	[292]
5	Neutral red C_15_H_17_N_4_ 288.78 g mol^−1^ 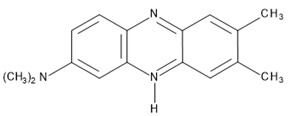	NR	[293]
6	Phenol red C_19_H_14_O_5_S 354.38 g mol^−1^ 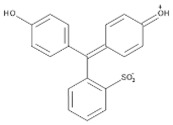	Ph R	[287]
7	4-Methyl phenol C_7_H_8_O 108.14 g mol^−1^ 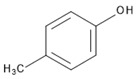	p-Cresol	[294]
8	Tetracycline C_22_H_24_N_2_O_8_ 444.435 g mol^−1^ 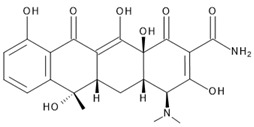	TC	[295]

**Table 13:** The performances of complex oxides having perovskite-type structure in the photodegradation of organic dyes.

Photocatalyst/ Photoelectrode	Dye	Light Source/Intensity	Experimental Conditions	Degradation Efficiency (%)	Ref.
SrTiO_3_	Rhodamine B (~5 ppm)	Ultraviolet (UV) light (200–400 nm)/3 × 15 W tubes	100 mg photocatalyst; Irradiation time: 1.3 h	100	[301]
SrTiO_3_	Rhodamine B (5 ppm)	UV light/15 W	226 mg/L photocatalyst; 20 °C; Irradiation: 4.5 h	60	[302]
SrTiO_3_	Rhodamine B (~5 ppm)	Visible (Vis) light (λ > 420 nm)/300 W	100 mg photocatalyst in 100 mL; Irradiation time: 6 h	<50	[304]
Fe-doped SrTiO_3_				~85	
Nb-doped SrTiO_3_	Rhodamine B (10 ppm)	Vis light (λ > 420 nm)	Irradiation time: 3 h	~50	[323]
KNbO_3_	Rhodamine B (40 ppm)	UV light/300 W	30 mg photocatalyst in 200 mL; Irradiation time: 4 h	71	[324]
NaNbO_3_	Rhodamine B (2.5 ppm)	UV light/300 W	Irradiation time: 1 h	72	[325]
LaCoO_3_	Rhodamine B (2 ppm)	UV light/500 W	10 mg photocatalyst; 35 °C; Irradiation time: 0.8 h	~100	[305]
GdFeO_3_	Rhodamine B (10 ppm)	Vis light (λ > 400 nm)/150 W	100 mg photocatalyst in 100 mL; Irradiation time: 3 h	~90	[326]
SmFeO_3_				~95	
BiFeO_3_	Rhodamine B (10 ppm)	Vis light/100W	300 mg photocatalyst; Irradiation time: 3 h	>30	[327]
BiFeO_3_	Rhodamine B (~5 ppm)	Vis light (λ > 420 nm)/500 W	100 mg photocatalyst in 50 mL; Irradiation time: 6 h	78	[328]
BiFeO_3_	Rhodamine B (10 ppm)	Vis light (λ > 420 nm)/300 W	50 mg photocatalyst in 50 mL; Irradiation time: 6 h	~60	[329]
Gd-doped BiFeO_3_	Rhodamine B (5 ppm)	Vis light (λ > 420 nm)/500 W	40 mg photocatalyst in 40 mL; Irradiation time: 2 h	94	[309]
LaFeO_3_	Rhodamine B (1000 ppm)	Vis light (λ > 400 nm)/150 W	100 mg photocatalyst in 100 mL; Irradiation time: 3 h	100	[316]
LaFeO_3_	Rhodamine B (10 ppm)	Vis light (λ > 400 nm)/150 W	100 mg photocatalyst in 100 mL; Irradiation time: 3 h	~96	[326]
LaFeO_3_	Rhodamine B (~5 ppm)	Vis light (λ > 400 nm)/500 W	10 mg photocatalyst; RT; Irradiation time: 2 h	76	[315]
LaFeO_3_	Rhodamine B (1000 ppm)	Vis light (λ > 400 nm)/150 W	100 mg in 100 mL; Irradiation time: 12 h	93	[316]
Ag/LaFeO_3_	Rhodamine B (10 ppm)	UV Vis light/125 W	100 mg photocatalyst; RT; Irradiation time: 2 h	92.8	[330]
SrTiO_3_	Methyl orange (10 ppm)	UV light/15 W	75 mg photocatalyst; RT; Irradiation time: 3 h	100	[303]
Nb-doped SrTiO_3_	Methyl orange (10 ppm)	Vis light (λ > 420 nm)	Irradiation time: 3h	~40	[323]
LaCoO_3_	Methyl orange (100 ppm)	Vis light	100 mg photocatalyst in 100 mL; Irradiation time: 2h	~60	[306]
LaCoO_3_	Methyl orange	UV light/30 W	Irradiation time: 1.6h	89	[331]
BiFeO_3_	Methyl orange (15 ppm)	UV-Vis light/300 W	30 mmol/L photocatalyst; Irradiation time: 8 h	>90	[307]
BiFeO_3_	Methyl orange (15 ppm)	Vis light/300 W	30 mmol/L photocatalyst; Irradiation time: 16 h	>90	[307]
BiFeO_3_	Methyl orange (5 ppm)	Vis light (λ > 420 nm)/300 W	200 mg photocatalyst; RT; Irradiation time: 4 h	>30	[38]
LaFeO_3_	Methyl orange (10 ppm)	Vis light(λ > 420 nm)/500 W	RT; Irradiation time: 4 h	>90	[317]
Nb-doped SrTiO_3_	Methylene blue (10 ppm)	Vis light (λ > 420 nm)	Irradiation time: 1.3 h	~85	[323]
KNbO_3_	Methylene blue (~13 ppm)	Vis light (λ > 420 nm)/180 mW/cm^2^	Irradiation time: 2 h	~50	[332]
NaNbO_3_	Methylene blue	UV light (306 nm)/1mW/cm^2^	Irradiation time: 24 h	~15	[320]
LaCoO_3_	Methylene blue (10 ppm)	UV light/30 W	200 mg/L photocatalyst; Irradiation time: 1.6 h	87	[333]
SrFeO_3_	Methylene blue (~4 ppm)	Vis light/8 W	Irradiation time: 12 h	100	[321]
LaFeO_3_	Methylene blue (10 ppm)	Vis light(λ>420 nm)/500 W	RT; Irradiation time: 4 h	93.8	[317]
Li-doped LaFeO_3_	Methylene blue (~31 ppm)	UV Vis light/250 W	100 mg photocatalyst in 50 mL; Irradiation time: 1 h	45.7	[334]
BiFeO_3_	Congo red (20 ppm)	Vis light (λ > 420 nm)/500 W	2 g/L photocatalyst; RT; Irradiation time: 3 h	~40	[335]
BiFeO_3_	Congo red (10 ppm)	Vis light (λ > 400 nm)/500 W	RT; Irradiation time: 4 h	~15	[336]
Ba-doped BiFeO_3_	Congo red (100 ppm)	Vis light/500 W	Irradiation time: 120 min	~30	[337]
Mn-doped BiFeO_3_	Congo red	Vis light(λ > 400 nm)/ 500 W	RT; Irradiation time: 2 h	~40	[338]
LaCoO_3_	Neutral red	UV light/30 W	Irradiation time: 0.6 h	88	[331]
La-doped BiFeO_3_	Phenol red (3.5 ppm)	Vis light (λ > 400 nm)/300 W	100 mg photocatalyst; RT; Irradiation time: 2 h	90.1	[333]
LaFeO_3_	4-methyphenol (10 ppm)	Vis light/1500 W	1200 mg photocatalyst; Irradiation time: 6 h	>90	[314]
Ca-doped LaFeO_3_				~70	
Fe-doped SrTiO_3_	Tetracycline (10 ppm)	Vis light (λ > 420 nm)/300 W	100 mg photocatalyst in 100 mL; Irradiation time: 1.3h	~71.6	[289]
